# TGF-β1 facilitates gallbladder carcinoma metastasis by regulating FOXA1 translation efficiency through m^6^A modification

**DOI:** 10.1038/s41419-024-06800-9

**Published:** 2024-06-17

**Authors:** Zhenheng Wu, Qiming Ke, Lei Jiang, Haijie Hong, Wei Pan, Wen Chen, Xiahenazi Abudukeremu, Feifei She, Yanling Chen

**Affiliations:** 1 Department of Hepatobiliary Surgery and Fujian Institute of Hepatobiliary Surgery, Fujian Medical University Union Hospital, Fuzhou, Fujian, 350001 China; 2 Fujian Medical University Cancer Center, Fuzhou, Fujian, 350122 China; 3 Key Laboratory of Gastrointestinal Cancer (Fujian Medical University), Ministry of Education, Fujian Medical University, Fuzhou, Fujian, 350122 China; 4 Fujian Key Laboratory of Tumor Microbiology, Department of Medical Microbiology, Fujian Medical University, Fuzhou, Fujian, 350122 China

**Keywords:** Gall bladder cancer, RNA modification

## Abstract

TGF-β1 plays a pivotal role in the metastatic cascade of malignant neoplasms. N6-methyladenosine (m^6^A) stands as one of the most abundant modifications on the mRNA transcriptome. However, in the metastasis of gallbladder carcinoma (GBC), the effect of TGF-β1 with mRNA m^6^A modification, especially the effect of mRNA translation efficiency associated with m^6^A modification, remains poorly elucidated. Here we demonstrated a negative correlation between FOXA1 and TGF-β1 expression in GBC. Overexpression of FOXA1 inhibited TGF-β1-induced migration and epithelial-mesenchymal transition (EMT) in GBC cells. Mechanistically, we confirmed that TGF-β1 suppressed the translation efficiency of FOXA1 mRNA through polysome profiling analysis. Importantly, both in vivo and in vitro experiments showed that TGF-β1 promoted m^6^A modification on the coding sequence (CDS) region of FOXA1 mRNA, which was responsible for the inhibition of FOXA1 mRNA translation by TGF-β1. We demonstrated through MeRIP and RIP assays, dual-luciferase reporter assays and site-directed mutagenesis that ALKBH5 promoted FOXA1 protein expression by inhibiting m^6^A modification on the CDS region of FOXA1 mRNA. Moreover, TGF-β1 inhibited the binding capacity of ALKBH5 to the FOXA1 CDS region. Lastly, our study confirmed that overexpression of FOXA1 suppressed lung metastasis and EMT in a nude mice lung metastasis model. In summary, our research findings underscore the role of TGF-β1 in regulating TGF-β1/FOXA1-induced GBC EMT and metastasis by inhibiting FOXA1 translation efficiency through m^6^A modification.

## Introduction

The incidence rate of GBC, the most common malignant neoplasm of the biliary system, is 2.5 per 100,000 individuals, accounting for approximately two-thirds of all biliary system tumors [1, 2]. Curative resection is considered the most effective strategy for potentially curing GBC in early-stage patients [3, 4]. However, the highly metastatic nature of GBC often leads to the loss of surgical opportunities for the majority of patients at the time of diagnosis, resulting in a dismal prognosis [3, 4]. Extensive research has highlighted the significance of epithelial-mesenchymal transition (EMT) as one of the important drivers of malignant tumor metastasis [5, 6]. Nevertheless, the precise mechanistic details regarding the regulation of EMT in the context of GBC and its role in promoting metastasis remain elusive.

In recent years, accumulating evidence has demonstrated that transcriptional growth factor beta 1 (TGF-β1) plays a crucial role in regulating tumor progression and EMT, and is referred to as an inducer of EMT [7, 8]. Studies have indicated that TGF-β1 expression is upregulated in GBC tissues, and is positively associated with distant metastasis in patients [9]. Moreover, TGF-β1 has been found to promote EMT in GBC cells [10]. However, the underlying mechanisms by which TGF-β1 induces EMT in GBC are still not fully elucidated. Therefore, the identification of specific targets mediating TGF-β1-induced EMT in GBC is pivotal to resolving this issue and holds significant implications in investigating the mechanisms underlying the propensity of GBC for metastasis.

Forkhead box A1 (FOXA1), known as a “pioneer factor,” interacts with chromatin to facilitate the relaxation of compact chromatin structure, thereby promoting transcriptional efficiency [11]. Studies have shown a close association between FOXA1 and EMT, although its specific functional mechanisms vary among different types of tumors and remain controversial. High expression of FOXA1 has been observed in acute myeloid leukemia [12], thyroid cancer [13], bladder cancer [14], esophageal cancer, and lung cancer [15], indicating its role in promoting tumor proliferation, invasion, and migration. Conversely, FOXA1 shows low expression and exhibits tumor-suppressive effects in pancreatic cancer [16] and nasopharyngeal cancer [17] by inhibiting proliferation and EMT. However, the role of FOXA1 in GBC has not been reported. Additionally, research has shown that TGF-β1 can induce the proliferation and differentiation of chondrocytes by promoting FOXA1 expression [18]. Conversely, TGF-β1 can mediate EMT in lung cancer by suppressing FOXA1 expression [19]. Therefore, it is hypothesized that FOXA1 may play an important role in TGF-β1-induced EMT in GBC. However, the mechanisms through which TGF-β1 regulates FOXA1 expression are still unclear.

The m^6^A methylation modification of RNA refers to the process in which the methyltransferase complex METTL3/METTL14/WTAP (“writers”) transfers the methyl group from S-adenosylmethionine to the N6 position of the target RNA’s adenine residue. Conversely, the demethylases (“erasers”) FTO/ALKBH5 play opposite roles, collectively regulating the m^6^A methylation modification level on cellular RNA [20–22]. Subsequently, m^6^A-modified RNA can be recognized by m^6^A methylation reader proteins (“readers”) [23–25], thereby influencing various processes including alternative splicing, structural changes, nuclear export, stability, and translation of m^6^A-modified RNA [26–30]. In the classical TGF-β signaling pathway, SMAD2/3 serves as the core complex through which TGF-β exerts its effects [31, 32]. Bertero et al., utilizing proteomic sequencing, discovered that SMAD2/3 interacts with the m^6^A methyltransferase complex in pluripotent stem cells [33], playing a regulatory role in the recruitment process of the m^6^A methyltransferase complex to target RNA [34].

This study sought to explore the effects of TGF-β1 on FOXA1 expression in GBC cells and to confirm FOXA1’s specific contributions to TGF-β1-driven EMT and migration in these cells. It aimed to delineate the molecular pathways through which TGF-β1 influences FOXA1 expression via m^6^A modification. Furthermore, in vivo experiments and the analysis of clinical GBC tissue specimens were conducted to assess FOXA1 expression and its association with clinical pathological features, reinforcing the significance of FOXA1 in GBC. The findings from this study would enhance our understanding of the mechanisms driving GBC metastasis and offer new perspectives for targeted treatment strategies in GBC.

## Materials and methods

### Clinical samples and cell lines

Clinical samples were collected according to the approved protocol by the Ethics Committee of the Medical College, Fujian Medical University, China. And written informed consent was obtained from all participants. 80 paraffin-embedded GBC tumor specimens and matched benign tissues were obtained from Fujian Medical University Union Hospital, China, spanning the years 2013 to 2022. All tumors were confirmed as GBC by the Department of Pathology. Patients did not receive any radiotherapy or chemotherapy prior to tumor resection. Human GBC cell lines, GBC-SD (purchased from the Shanghai Institutes for Biological Sciences, China) and NOZ (obtained from the Health Science Research Resources Bank, Japan). Both cell lines underwent mycoplasma detection and STR cell identification to ensure their authenticity and purity. GBC cells were maintained in DMEM (Hyclone, USA) supplemented with 10% fetal bovine serum (Hyclone, USA). All cells were cultured in a humidified incubator at 37 °C with 5% CO2.

### Reverse transcription and real-time PCR (RT-qPCR)

Total RNA was extracted from GBC tissues or cells using TRIzol (Invitrogen) according to the manufacturer’s instructions. Reverse transcription was performed using the All-In-One 5×RT MasterMix (Abm, Canada). Real-time PCR was carried out using the Fast Start Universal SYBR Green Master Mix (Roche, Basel, Switzerland) and fluorescence was measured using the ABI 7500 real-time system (Applied Biosystems, Life Technologies), following the manufacturer’s instructions. GAPDH was used as an internal control. Data analysis was performed using the 2^−ΔΔCt^ method. The detailed primer sequences are provided in Table 1.

**Table 1:** Detailed information of the primer sequences in this study.

Gene	Primer sequence
FOXA1	F 5′-CACTGCAATACTCGCCTTAGCG-3′
	R 5′-AGGACGGGTCTGGAATACAC-3′
METTL3	F 5′-CTGCAACGCATCATTCGGAC-3′
	R 5′-AGACCCTGGTTGAAGCCTTG-3′
METTL14	F 5′-TGGACCTTGGAAGAGTGTGTT-3′
	R 5′-GTGCTACGCTTCACAGTTCC-3′
WTAP	F 5′-AATCCAGTACCTCAAGCAAGTC-3′
	R 5′-TGTCTTTAGTCTGTTCCAGTTCAC-3′
FTO	F 5′-CTGGAAGCACTGTGGAAGAAG-3′
	R 5′-GCAAGGATGGCAGTCAAGATT-3′
ALKBH5	F 5′-CTCTTCAGCCAGGACGAGTG-3′
	R 5′-CCGTAAGTGTAGCCTTCGCC-3′
U2	F5′-TTGGGAATTCTCAAGTGTAGTATCTGTTCTTAT-3′
	R 5′-AAGGCGAATTCGCGATGCGCTCGCCTTCGCGCCC-3′
S14	F 5′-GGCAGACCGAGATGAATCCTC-3′
	R 5′-CAGGTCCAGGGGTCTTGGTCC-3′
GAPDH	F 5′-GGTGTGAACCATGAGAAGTATGA-3′
	R 5′-GAGTCCTTCCACGATACCAAAG-3′

### Western blot analysis and antibodies

Protein blotting analysis was performed as previously described [35]. The following primary antibodies were purchased from Abcam: rabbit anti-TGF-β1 (ab215715), rabbit anti-FOXA1 (ab170933), rabbit anti-METTL3 (ab195352), rabbit anti-METTL14 (ab300104), rabbit anti-WTAP (ab195380), rabbit anti-FTO (ab126605), rabbit anti-ALKBH5 (ab195377), and rabbit anti-Flag (ab205606). The following primary antibodies were purchased from Cell Signaling Technology: rabbit anti-E-Cadherin (#3195), rabbit anti-N-Cadherin (#13116), rabbit anti-Vimentin (#5741), rabbit anti-Snail (#3879), rabbit anti-Slug (#9585), and secondary antibody (#7074). Rabbit anti-GAPDH (#5174) was used as a protein loading control for cytoplasmic proteins. The antibody-antigen complex signal was detected using the ECL substrate kit (Advansta, USA), according to the manufacturer’s instructions.

### Generation of lentiviral constructs and transfection

Lentiviral constructs overexpressing Flag-tagged FOXA1-GFP (Flag-FOXA1) and control vectors (Vector 1), as well as Flag-tagged ALKBH5-GFP (Flag-ALKBH5) and control vectors (Vector 2), were obtained from GENE (Shanghai, China). Stable transfectants of GBC-SD and NOZ cells overexpressing FOXA1 (Flag-FOXA1) and ALKBH5 (Flag-ALKBH5) were generated by transfection with lentiviral constructs followed by selection with puromycin for 2 weeks, according to the manufacturer’s instructions. Lentivector-mediated short-hairpin FOXA1 (sh-FOXA1) and non-targeting plasmids (sh-Scr) were designed and synthesized by GENE (Shanghai, China). Stable transfer GBC cells with FOXA1 knockdown were generated in the same way.

### Chemical inhibitors

The following exogenous drugs used in this study were purchased from MCE (MedChemExpress, USA): TGF-β1 (HY-P7118), MG132 (HY-13259), Cycloheximide (CHX, HY-12320), Actinomycin D (Act-D, HY-17559), FB23 (HY-137187), and STM2457 (HY-134836). AZ12601011 (HY-122856), UC2288 (HY-112780), 10058-F4 (HY-12702).

### Immunohistochemistry analysis (IHC)

Immunohistochemistry analysis was performed as previously described [36]. Primary antibodies specific for TGF-β1 (1:500, Abcam), FOXA1 (1:400, Abcam), E-Cadherin (1:200, #3195), N-Cadherin (1:200, #13116), and Vimentin (1:300, #5741) were incubated overnight at 4 °C. After the incubation, the slides were washed and then stained using a diaminobenzidine (DAB) detection kit (Gene Tech, China) followed by counterstaining with hematoxylin. Images of five random fields per section were captured using a light microscope (Olympus VS200, Tokyo, Japan). The immunohistochemical evaluation was quantified based on the product of staining intensity and the percentage of positive cells. The percentage of positively stained cells was scored on a scale from 0 to 4 (0 = 0–10%; 1 = 11–25%; 2 = 26–50%; 3 = 51–75%; 4 = 76–100%), and the staining intensity was rated from 0 to 3 (0 = negative staining; 1 = weak; 2 = moderate; 3 = strong).

### Xenograft models

Female athymic BALB/c nude mice (4-6 weeks old, n = 7) were purchased from SIPEIFU (Beijing, China). All mice were housed under specific pathogen-free conditions in accordance with the approved protocol of the Ethical Committee of the Medical College, Fujian Medical University (Approval No: IACUC FJMU 2023-0140).

GBC cells (1 × 10^6^ NOZ cells) suspended in 100 μl sterile PBS were subcutaneously transplanted into the right hind limb of mice to establish a subcutaneous xenograft model. Once subcutaneous tumors formed, the average tumor size was measured, and mice were randomly divided into experimental groups. The experimental group received peritumorial injections of TGF-β1 (4 mg/kg) every 7 days, while the control group received injections of saline. Mice were monitored weekly, and tumor volume was calculated using the formula: (length × width^2^)/2. After 4 weeks of TGF-β1 injection, mice were euthanized, and xenograft specimens were surgically excised and weighed. For lung metastasis model, mice were randomly divided into two experimental groups (4-6 weeks old, n = 6). GBC cells (NOZ) overexpressing FOXA1 (Flag-FOXA1 group) and the corresponding vector control group were injected into mice via the tail vein with 1 × 10^6^ cells suspended in 100 μl sterile PBS to establish a lung metastasis model. After 4 weeks of cell injection, the progression of cell metastasis was observed by intraperitoneal injection of D-luciferin potassium salt (Synchem, Germany) and imaging using the IVIS-100x system (Caliper Life Sciences, USA). Subsequently, mice were euthanized, and lung metastasis specimens were surgically excised and the number of metastatic lung nodules was counted.

### mRNA stability

Actinomycin D (final concentration 5 μg/ml) was added to the cells. After incubation for a specified time, total RNA was extracted from GBC cells using TRIzol for RT-qPCR validation.

### Protein stability

Cycloheximide (CHX, final concentration 50 μg/ml) or MG132 (10 μM) was added to the cells. After incubation for a specified time, protein blotting was performed to examine the protein levels of FOXA1. The quantification of FOXA1 was performed by normalizing its band intensity against the internal control, GAPDH. The optical density of the FOXA1 bands at two, four, and six hours was normalized to the value at time zero for each respective time period. The percentage of optical density was calculated, with the ratio at time zero arbitrarily set as 100%.

### Subcellular fractionation

Nuclear and cytoplasmic fractions were separated using a nuclear-cytosol fractionation kit (Norgen Biotek Corp.), according to the manufacturer’s instructions. S14 was used as a loading control for the cytoplasmic fraction, while U2 snRNA served as a loading control for the nuclear fraction. RT-qPCR was performed to validate the distribution of FOXA1 mRNA in the nuclear and cytoplasmic fractions.

### Methylated RNA immunoprecipitation (MeRIP-qPCR)

MeRIP-qPCR was performed using the EpiQuik™ CUT&RUN m^6^A RNA enrichment kit (Epigentek, USA), following the manufacturer’s instructions. Briefly, 10 μg of total RNA, affinity beads, and m^6^A antibody were mixed in a 0.2 ml PCR tube and incubated at room temperature with rotation for 90 min. The enriched RNA was then released, purified, and eluted. The eluted RNA was subjected to RT-qPCR, and the enrichment of the corresponding m^6^A fragments in each sample was calculated by normalizing to the input group (625 ng of RNA as the input). The primer sequence for the m^6^A site of FOXA1 is provided in Table 2.

**Table 2:** Detailed information of the primer sequences in this study.

Gene	Primer sequence
FOXA1 984	F 5′-CCACTCGCTGTCCTTCAATG-3′
	R 5′-TAGCAGCCGTTCTCGAACAT-3′
FOXA1 1277	F 5′-CCGACTCGCCCCTCCATC-3′
	R 5′-CCCACTGTGGTCCAGAGTCT-3′
FOXA1 1524	F 5′- TTCAACCACCCGTTCTCCAT-3′
	R 5′- GTGCCTGTTCGTATGCCTTG-3′
FOXA1 1705	F 5′-ACCCGTCCTAAACACTTCCT-3′
	R 5′-TGTGGTTTTGTTTGCTGTTGATTT-3′
FOXA1 1778	F 5′-CACACAAACCAAACCGTCAAC-3′
	R 5′-AGTCTTTTGCACTGGGGGAA-3′
FOXA1 1861	F 5′- ACCGTCAACAGCATAATAAAATCC-3′
	R 5′- GGTTTGGGGTTGTCTTTGTAGTA-3′

### RNA immunoprecipitation (RIP RT-qPCR)

GBC cells with or without overexpression of ALKBH5 were seeded in 10 cm culture dishes at a confluency of 80%-90% and harvested by trypsin digestion. Magnetic beads were then coupled with Flag antibody or IgG antibody, and antigen capture was performed according to the specific protocol of the RNA immunoprecipitation kit (Geneseed, Guangzhou, China). The enriched RNA was subsequently released, purified, and eluted for validation by RT-qPCR.

### Dual-luciferase reporter assay

GV238 plasmid containing the FOXA1 promoter −2000/+100, GV272 plasmids containing the wild-type (WT) and mutant (MUT) coding sequence (CDS) region of FOXA1, and GV272 plasmids containing the wild-type and mutant 3’ untranslated region (3’UTR) of FOXA1 were obtained from GENE (Shanghai, China). After transfection into GBC cells for 48 h, firefly luciferase (F-luc) activity was measured using the Dual-Luciferase Reporter Assay System (Promega) according to the manufacturer’s instructions. Renilla luciferase (R-luc) served as an internal control.

### Polysome profiling

CHX (final concentration 100 μg/ml) was added to 1 × 10^7^ GBC-NOZ cells and incubated for 15 min. Cells were collected by trypsin digestion in the presence of CHX (100 μg/ml). Subsequently, 1 ml of polysome lysis buffer was added, and cells were lysed on ice for 30 min, followed by centrifugation at 15,000 rpm for 15 min at 4 °C. The supernatant was collected and overlaid on a linear sucrose gradient (10%-45%) and centrifuged at 36,000 rpm for 3 h. After centrifugation, 18 fractions were collected, and RNA was extracted from each fraction using Trizol. The content of FOXA1 mRNA in each fraction was then analyzed by RT-qPCR.

### Statistical analysis

In this study, all statistical analyses were performed using SPSS 19.0 software package (SPSS Inc. Chicago, USA), GraphPad Prism 8 software (GraphPad, USA), and Microsoft Office 2019 EXCEL (USA). Two-group comparisons were performed using t-test, while differences among multiple groups were analyzed by analysis of variance (One way ANOVA). Chi-square analysis and Pearson correlation analysis were used for clinical pathological data analysis. All data were presented as mean ± standard error of the mean (SEM) from independent replicates (n ≥ 3). Differences with a *p* value less than 0.05 were considered statistically significant.

## Results

### TGF-β1 and FOXA1 expression are negatively correlated in GBC

**Fig. 1:**
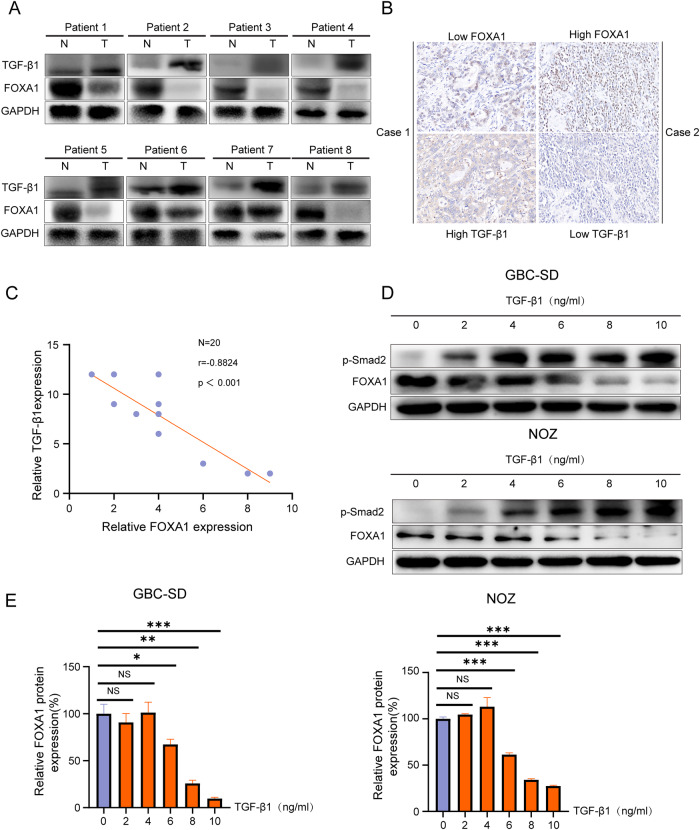
The relationship between TGF-β1 and FOXA1 in GBC. **A** Western blot analysis of TGF-β1 and FOXA1 expression in 8 cases of GBC tissues and corresponding adjacent non-cancerous tissues. **B** Immunohistochemistry analysis of TGF-β1 and FOXA1 expression in 20 GBC tissues. **C** Pearson correlation analysis of TGF-β1 protein expression and FOXA1 protein expression in a batch of 20 GBC cases. **D**, **E** Western blot analysis of p-Smad2 and FOXA1 expression in GBC cells stimulated with different concentrations of TGF-β1 after 48 h. Error bars represent the mean (n = 3) ±SEM. ^NS^*p* > 0.05, *p < 0.05, **p < 0.01, ***p < 0.001.

Western blot analysis showed that intracellular TGF-β1 expression was higher in GBC tissues compared to adjacent non-cancer tissues, while FOXA1 expression was higher in adjacent non-cancer tissues than in GBC tissues in 8 cases of GBC (Fig. 1A). Immunohistochemistry analysis of consecutive pathological slices from 20 cases of GBC showed that high TGF-β1 expression correlated with low FOXA1 expression, and the reverse was also true (Fig. 1B). Correlation analysis revealed a negative correlation between intracellular TGF-β1 expression and FOXA1 expression in GBC (Fig. 1C).

GBC cells GBC-SD and NOZ were treated with different concentrations of exogenous TGF-β1. After 48 h, Western blot analysis was performed to detect p-Smad2 and FOXA1 expression levels. The results showed that exogenous TGF-β1 led to an up-regulation of p-SMAD2 expression in GBC cells, which escalated as the concentration of TGF-β1 increased (Fig. 1D). Moreover, compared to the control group treated with 0 ng/ml TGF-β1, there was no significant difference in the 2 ng/ml and 4 ng/ml TGF-β1 groups. However, TGF-β1 expression significantly decreased in the 6 ng–10 ng/ml TGF-β1 groups, showing a concentration-dependent effect (Fig. 1D, E). These results not only corroborate the observations from the tissue studies but also indicates that TGF-β1 actively participates in modulating FOXA1 levels. Overall, these comprehensive analysis from both endogenous and exogenous perspectives conclusively demonstrates the inhibitory effect of TGF-β1 on FOXA1 protein expression in GBC.

### The role of FOXA1 in TGF-β1-induced GBC cell migration and EMT

During the EMT process, the tight junctions and adherens junctions between epithelial cells gradually disappear, while acquiring migratory and invasive capabilities [37]. EMT markers, such as E-cadherin, N-cadherin, vimentin, Snail and Slug, play important roles in regulating the invasion and metastasis of tumor cells [38, 39]. It is known that TGF-β1 promotes GBC cell migration and induces EMT [40], but the role of FOXA1 in GBC is not clear. By knocking down FOXA1 in GBC-SD and NOZ cells, we explored the impact of FOXA1 on the EMT in GBC cells (Supplementary Fig. S1A). Transwell assays indicated a significant increase in cell migration in the sh-FOXA1 group compared to the control sh-Scr group (Supplementary Fig. S1B, C). Similarly, wound healing assays demonstrated that migration was significantly enhanced in the sh-FOXA1 group relative to the sh-Scr group (Supplementary Fig. S1D, E). Western blot analysis revealed that the sh-FOXA1 group exhibited a marked decrease in E-cadherin expression and an increase in N-cadherin, vimentin, and the EMT-related transcription factors Snail and Slug, compared to the sh-Scr group (Supplementary Fig. S1F). These findings suggest that FOXA1 plays a role in inhibiting migration and EMT in GBC cells.

**Fig. 2:**
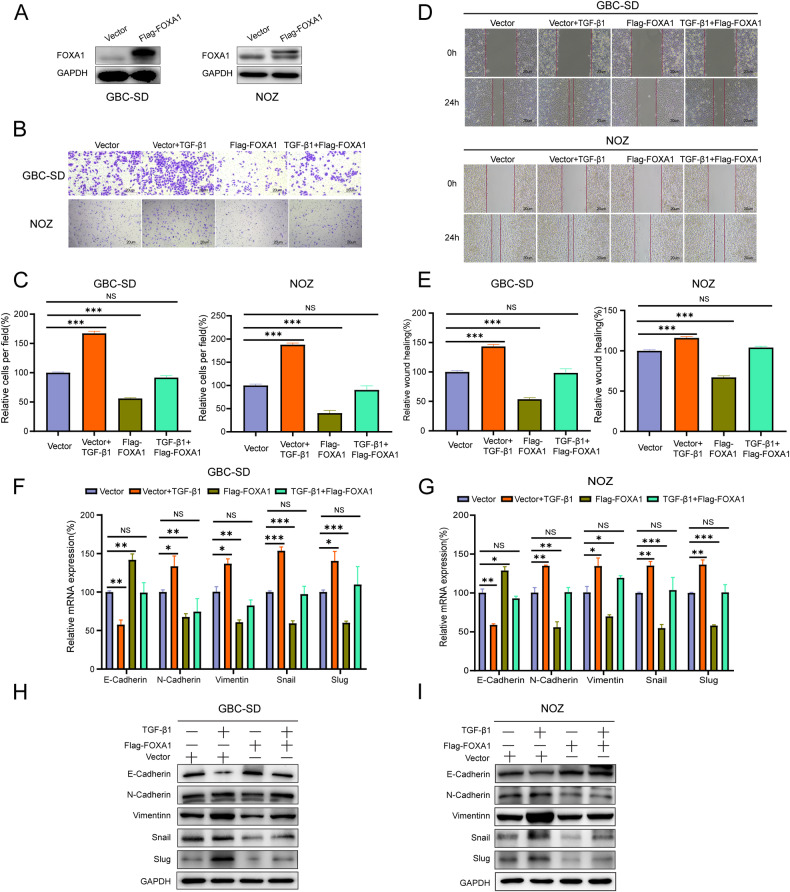
The role of FOXA1 in TGF-β1-induced GBC cell migration. **A** Western blot analysis of the overexpression efficiency of FOXA1. **B**, **C** Transwell assay to determine the changes in the migratory capacity of GBC cells after the addition of TGF-β1 and overexpression of FOXA1. **D**, **E** Wound healing assay to assess the changes in the migratory capacity of GBC cells after the addition of TGF-β1 and overexpression of FOXA1. **F**, **G** RT-qPCR to examine the changes in the RNA levels of EMT-related markers in GBC cells after the addition of TGF-β1 and overexpression of FOXA1. **H**, **I** Western blot analysis to determine the changes in the protein levels of EMT-related markers in GBC cells after the addition of TGF-β1 and overexpression of FOXA1.Error bars represent the mean (n = 3) ±SEM. ^NS^*p* > 0.05, *p < 0.05, **p < 0.01, ***p < 0.001.

GBC-SD and NOZ GBC cells were infected with lentivirus overexpressing FOXA1 to establish stable overexpression cell lines (Flag-FOXA1 group), as well as cells transfected with empty vector (Vector group) as control (Fig. 2A). Transwell assay showed that the number of migrated cells was significantly increased in the Vector+TGF-β1 group compared to the Vector group, while it was significantly decreased in the Flag-FOXA1 group compared to the Vector group. There was no significant difference in the number of migrated cells between the Flag-FOXA1 + TGF-β1 group and the Vector group (Fig. 2B, C). Wound healing assay revealed that the number of migrated cells was significantly increased in the Vector+TGF-β1 group compared to the Vector group, while it was significantly decreased in the Flag-FOXA1 group compared to the Vector group. There was no significant difference in the number of migrated cells between the Flag-FOXA1 + TGF-β1 group and the Vector group (Fig. 2D, E). Collectively, these results suggest that FOXA1 inhibits TGF-β1-induced GBC cell migration.

RT-qPCR and Western blot analysis demonstrated the role of FOXA1 in TGF-β1-induced EMT in GBC cells. The results showed that in GBC-SD and NOZ cells, TGF-β1 treatment significantly downregulated E-cadherin expression and upregulated N-cadherin, vimentin, and EMT-related transcription factors Snail and Slug expression levels compared to the Vector group. Overexpression of FOXA1 significantly upregulated E-cadherin expression and downregulated N-cadherin, vimentin, Snail and Slug expression levels compared to the Vector group. Moreover, FOXA1 suppressed the downregulation of E-cadherin and the upregulation of N-cadherin, Vimentin, Snail and Slug induced by TGF-β1 (Fig. 2F–I). These results indicate that FOXA1 inhibits TGF-β1-induced EMT in GBC cells.

### The effect of TGF-β1 on the transcription and translation of FOXA1 in GBC cells

Next, we explored the molecular mechanism by which TGF-β1 inhibits the protein expression of FOXA1 in GBC cells. The activation of the p-Smad2 signaling pathway is a critical mechanism for the action of TGF-β1 [41]. As anticipated, with increasing concentrations of exogenous TGF-β1, there was a corresponding rise in p-Smad2 protein expression (Fig. 1D). However, when we introduced the SMAD2 phosphorylation inhibitor AZ12601011 [42], it only weakly mitigated the suppression of FOXA1 protein expression by TGF-β1 (Supplementary Fig. S2A). This suggests that the p-SMAD2 pathway may not be the primary route through which TGF-β1 inhibits FOXA1 protein expression in GBC cells. Additionally, while TGF-β1 is known to suppress cell cycle progression through the regulation of cyclin-dependent kinase (CDK) inhibitors like p21 and c-Myc [43], the addition of the p21 inhibitor UC2288 [44] and the c-Myc inhibitor 10058-F4 [45] did not significantly counteract the TGF-β1-induced inhibition of FOXA1 protein expression (Supplementary Fig. S2B). This indicates that the cell cycle arrest induced by TGF-β1 does not significantly influence FOXA1 protein expression in GBC cells.

**Fig. 3:**
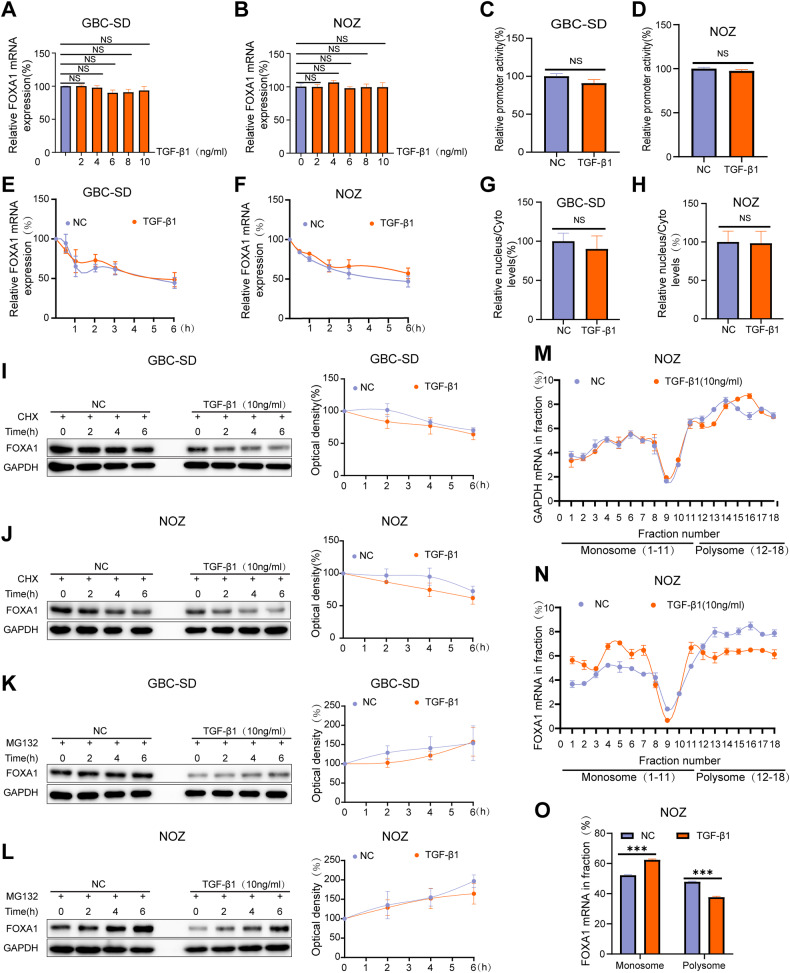
The effect of TGF-β1 on FOXA1 transcription and translation in GBC cells. **A**, **B** RT-qPCR analysis of the impact of different concentrations of TGF-β1 on FOXA1 mRNA. **C**, **D** Dual-luciferase reporter assay to assess the effect of TGF-β1 on FOXA1 promoter activity. **E**, **F** Act-D treatment for the corresponding time period followed by RT-qPCR to evaluate the influence of TGF-β1 on the mRNA half-life of FOXA1. **G**, **H** RT-qPCR analysis of the effect of TGF-β1 on the subcellular distribution of FOXA1 mRNA. **I**, **J** CHX treatment for the corresponding time period followed by Western blot analysis to examine the impact of TGF-β1 on the protein degradation rate of FOXA1. **K**, **L** MG132 treatment for the corresponding time period followed by Western blot analysis to assess the influence of TGF-β1 on the protein degradation rate of FOXA1. **M**, **N** The distribution of the housekeeping gene GAPDH mRNA and FOXA1 mRNA in each component. **O** The effect of TGF-β1 on the mRNA content of FOXA1 in the monosome and polysome. Error bars represent the mean (n = 3) ±SEM. ^NS^*p* > 0.05, *p < 0.05.

The effect of TGF-β1 on FOXA1 mRNA expression was determined using RT-qPCR. The results showed that TGF-β1 had no effect on FOXA1 mRNA expression in GBC cells GBC-SD and NOZ (Fig. 3A, B). Furthermore, a dual-luciferase reporter assay confirmed that TGF-β1 had no significant impact on the promoter activity of FOXA1 (Fig. 3C, D), indicating that TGF-β1 does not promote the transcription of FOXA1. To investigate the mRNA half-life of FOXA1, cells were treated with Act-D to block the transcription process. RT-qPCR confirmed that TGF-β1 did not alter the mRNA half-life of FOXA1 (Fig. 3E, F). RNA from nuclear and cytoplasmic fractions was separated, and RT-qPCR results showed that TGF-β1 did not affect the subcellular distribution of FOXA1 mRNA (Fig. 3G, H). Therefore, the reason for TGF-β1-induced inhibition of FOXA1 protein levels without altering FOXA1 mRNA levels may be due to an increased degradation rate of FOXA1 protein or a decrease in the translation efficiency of FOXA1 mRNA caused by TGF-β1.

To investigate the protein degradation rate of FOXA1, cells were treated with CHX to inhibit protein translation or MG132 to inhibit proteasome activity. Western blot analysis showed that TGF-β1 did not alter the protein degradation rate of FOXA1 (Fig. 3I–L). Polysome profiling analysis was performed to explore whether TGF-β1 leads to a decrease in the translation efficiency of FOXA1 mRNA. In GBC cells NOZ, fractions containing non-translated fragments (<40S), translational initiation fragments (including 40S, 60S, 80S monomers, and <80S), and actively translating polysomes (>80S) were separated. The distribution of the housekeeping gene GAPDH mRNA and FOXA1 mRNA in each component is shown respectively in Fig. 3M and Fig. 3N. Apparently, TGF-β1 increased the mRNA content of FOXA1 in the monomer fraction and decreased the mRNA content of FOXA1 in the actively translating polysome fraction (Fig. 3O), indicating that TGF-β1 inhibits the translation efficiency of FOXA1 mRNA. In summary, TGF-β1 inhibits FOXA1 protein levels without altering FOXA1 mRNA levels due to its inhibitory effect on the translation efficiency of FOXA1 mRNA.

### TGF-β1 promotes m^6^A modification of FOXA1 mRNA in GBC cells

**Fig. 4:**
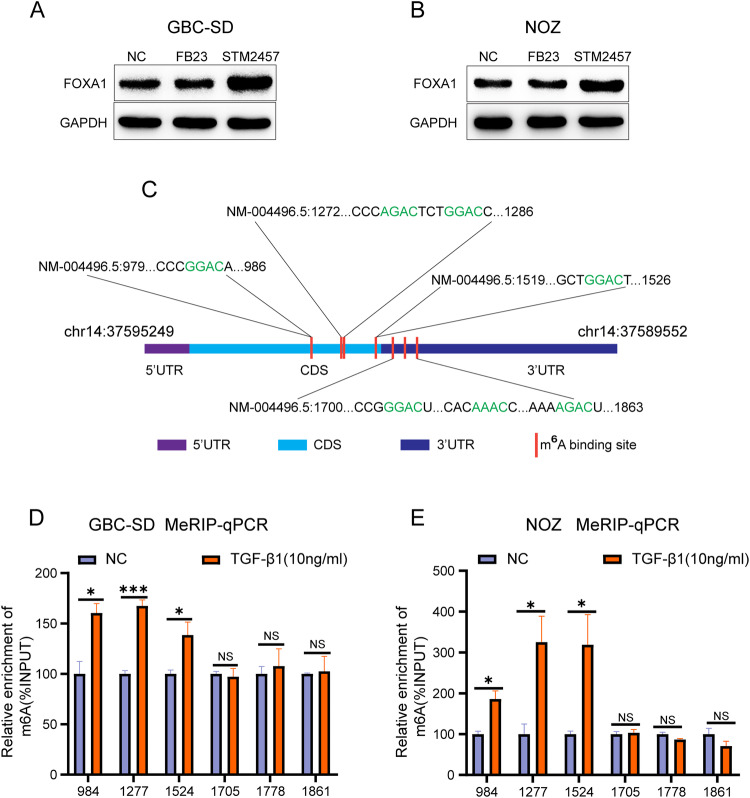
TGF-β1 promotes m^6^A modification of FOXA1 mRNA in GBC cells. **A**, **B** Western blot analysis of the effect of m^6^A modification inhibitors on FOXA1 protein expression. **C** Identification of m^6^A modification sites on FOXA1 mRNA. **D**, **E** MeRIP qPCR analysis to assess the impact of TGF-β1 on m^6^A modification of FOXA1 mRNA. Error bars represent the mean (n = 3) ±SEM. ^NS^*p* > 0.05, *p < 0.05, **p < 0.01, ***p < 0.001.

In eukaryotes, RNA modifications are among the factors that inhibit mRNA translation efficiency. m^6^A methylation is the most common chemical modification that occurs on RNA in eukaryotes. Therefore, TGF-β1 may inhibit the translation efficiency of FOXA1 mRNA by affecting m^6^A modification of FOXA1 mRNA. Following treatment of GBC cells GBC-SD and NOZ with the m^6^A demethylase FTO-targeting inhibitor FB23 and the m^6^A methyltransferase METTL3-targeting inhibitor STM2457, the results showed that FB23 did not alter the protein levels of FOXA1, while STM2457 significantly increased the protein levels of FOXA1 (Fig. 4A, B). Additionally, the addition of STM2457 can rescue the inhibitory effect of TGF-β1 on FOXA1 protein expression (Supplementary Fig. S3), indicating that m^6^A modification plays a critical role in the inhibition of FOXA1 protein expression by TGF-β1. The m^6^A modification sites on FOXA1 mRNA were predicted using the online database SARMP [46] (http://www.cuilab.cn/sramp). Within the highest confidence interval, there are four m^6^A methylation sites in the CDS region of FOXA1 mRNA and three m^6^A methylation sites in the 3’ UTR region (Fig. 4C). Methylated RNA immunoprecipitation (MeRIP) experiments revealed that TGF-β1 promotes m^6^A modification of the CDS region of FOXA1 mRNA, while it did not alter the m^6^A modification of the 3’ UTR region of FOXA1 mRNA (Fig. 4D, E).

### TGF-β1 promotes m^6^A modification of FOXA1 mRNA in GBC

**Fig. 5:**
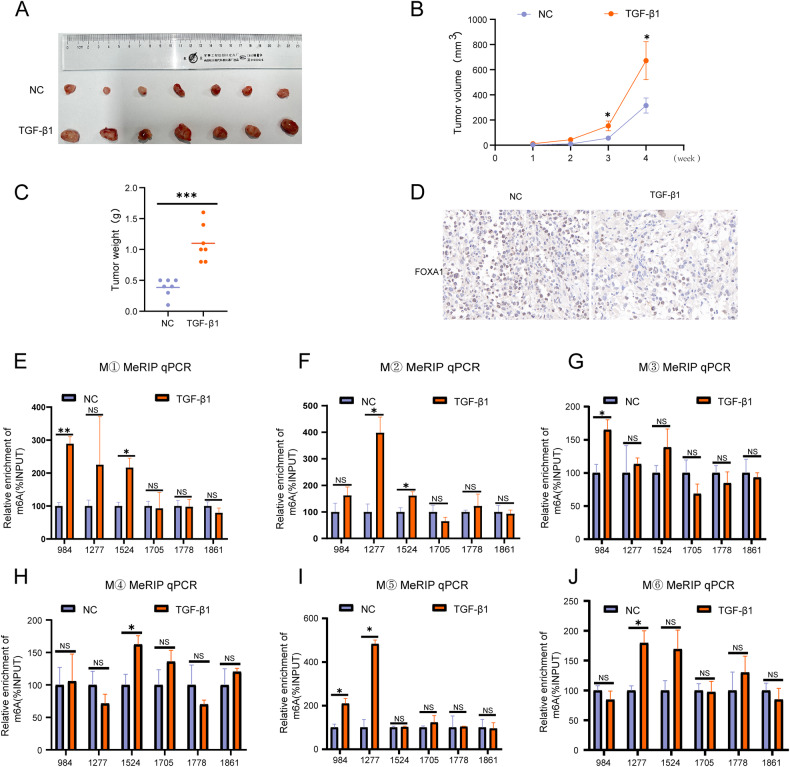
TGF-β1 promotes m^6^A modification of FOXA1 mRNA in GBC. **A** Effect of TGF-β1 on the size of subcutaneous tumors in GBC. Impact of TGF-β1 on the (**B**) volume and (**C**) weight of subcutaneous tumors in GBC. **D** Immunohistochemical analysis of the effect of TGF-β1 on FOXA1 protein expression in subcutaneous tumors of GBC. **E**–**J** MeRIP qPCR analysis to assess the impact of TGF-β1 on m^6^A modification of FOXA1 mRNA in subcutaneous tumors of GBC. Error bars represent the mean (n = 3) ±SEM. ^NS^*p* > 0.05, *p < 0.05, **p < 0.01, ***p < 0.001.

A subcutaneous xenograft model was established using human GBC cells (NOZ) in nude mice. Animals were euthanized 4 weeks after TGF-β1 injections. The results showed that TGF-β1 promoted the growth of subcutaneous GBC xenografts (Fig. 5A), as well as increased the volume and weight of the xenografts (Fig. 5B, C). Furthermore, TGF-β1 was found to inhibit the expression of FOXA1 in the subcutaneous tumors (Fig. 5D). Subsequently, the extracted RNA from subcutaneous tumors was subjected to MeRIP analysis, which revealed that TGF-β1 promoted m^6^A modification of FOXA1 mRNA in the CDS region, without altering m^6^A modification in the 3’ UTR region of FOXA1 mRNA (Fig. 5E–J).

### ALKBH5 is involved in the process of TGF-β1-promoted m^6^A modification of FOXA1 mRNA

**Fig. 6:**
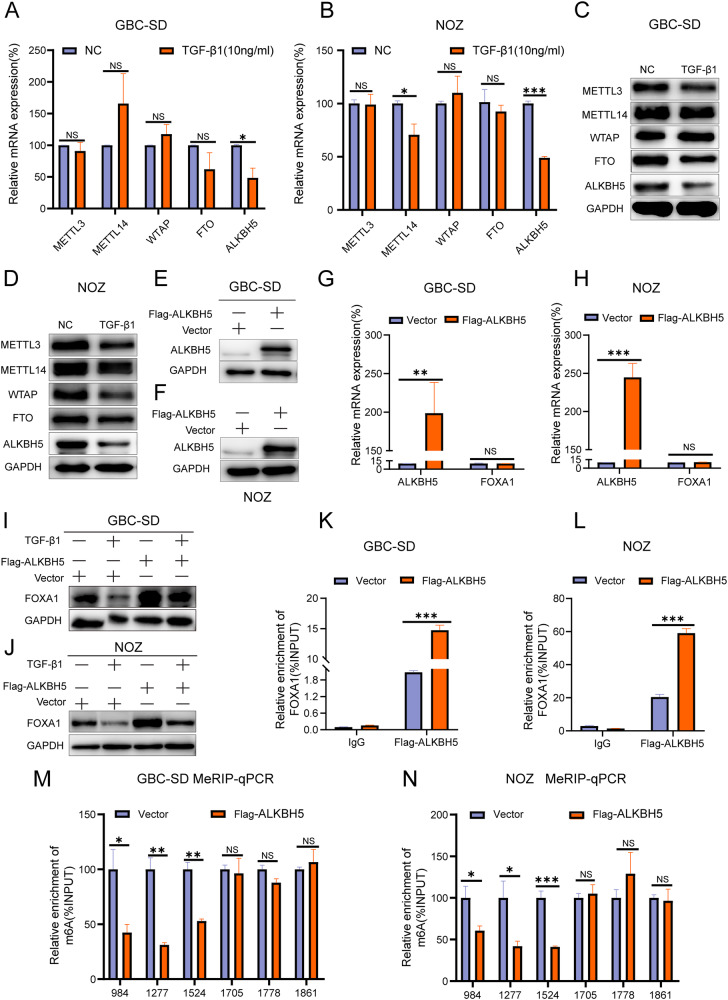
TGF-β1 inhibits FOXA1 expression by suppressing ALKBH5. **A**, **B** RT-qPCR analysis of the impact of TGF-β1 on the RNA expression of key m^6^A enzymes. **C**, **D** Western blot analysis of the effect of TGF-β1 on the protein expression of key m^6^A enzymes. **E**, **F** Western blot analysis of ALKBH5 overexpression. **G**, **H** RT-qPCR analysis of the effect of ALKBH5 overexpression on FOXA1 mRNA expression. **I**, **J** Western blot analysis of changes in FOXA1 protein after TGF-β1 induction and ALKBH5 overexpression. **K**, **L** RIP qPCR analysis of the interaction between ALKBH5 and FOXA1 mRNA. **M**, **N** MeRIP qPCR analysis of the impact of ALKBH5 overexpression on m^6^A modification of FOXA1 mRNA. Error bars represent the mean (n = 3) ±SEM. ^NS^*p* > 0.05, *p < 0.05, **p < 0.01, ***p < 0.001.

After TGF-β1 stimulation, changes in the main components of the m^6^A methyltransferase complex, including METTL3/METTL14/WTAP, and the demethylases ALKBH5 and FTO, were detected using RT-qPCR and Western blot analysis. RT-qPCR showed that TGF-β1 inhibited the mRNA levels of ALKBH5, while the impact on other m^6^A modification factors was not statistically significant (Fig. 6A, B). Western blot analysis showed that TGF-β1 decreased the protein levels of METTL3, FTO, and ALKBH5 (Fig. 6C, D). Previous experiments demonstrated that the FTO-targeting inhibitor FB23 did not alter the protein levels of FOXA1, while METTL3 was identified as a factor that promotes m^6^A modification. These findings suggest that TGF-β1 may promote m^6^A modification of FOXA1 mRNA by inhibiting the expression of the m^6^A demethylase ALKBH5.

To explore the role of ALKBH5 in TGF-β1-induced m^6^A modification of FOXA1 mRNA, a stable cell line of GBC cells overexpressing ALKBH5 (Flag-ALKBH5) was established using lentiviral constructs (Fig. 6E, F). RT-qPCR analysis showed that overexpression of ALKBH5 had no effect on the levels of FOXA1 mRNA (Fig. 6G, H). However, overexpression of ALKBH5 significantly increased the protein levels of FOXA1 and attenuated the inhibitory effect of TGF-β1 on FOXA1 protein (Fig. 6I, J), indicating that ALKBH5 is involved in the process of TGF-β1-mediated suppression of FOXA1 protein expression.

Further investigation was conducted to understand the regulatory mechanism of ALKBH5 on FOXA1. RNA immunoprecipitation (RIP) results showed that ALKBH5 can bind to FOXA1 mRNA (Fig. 6K, L). Additionally, MeRIP results demonstrated that overexpression of ALKBH5 reduced m^6^A modification levels in the CDS region of FOXA1 mRNA, while having no effect on m^6^A modification levels in the 3’ UTR region of FOXA1 mRNA (Fig. 6M, N). Additionally, RIP assay results demonstrate that ALKBH5 can bind to m^6^A modification sites in the CDS region of FOXA1 mRNA, but not to modification sites in the 3’ UTR (Supplementary Fig. S4). In summary, ALKBH5 is a key gene involved in TGF-β1-promoted m^6^A modification of FOXA1 mRNA.

### ALKBH5 regulates the m^6^A modification sites of FOXA1 mRNA contributing to the expression of FOXA1

**Fig. 7:**
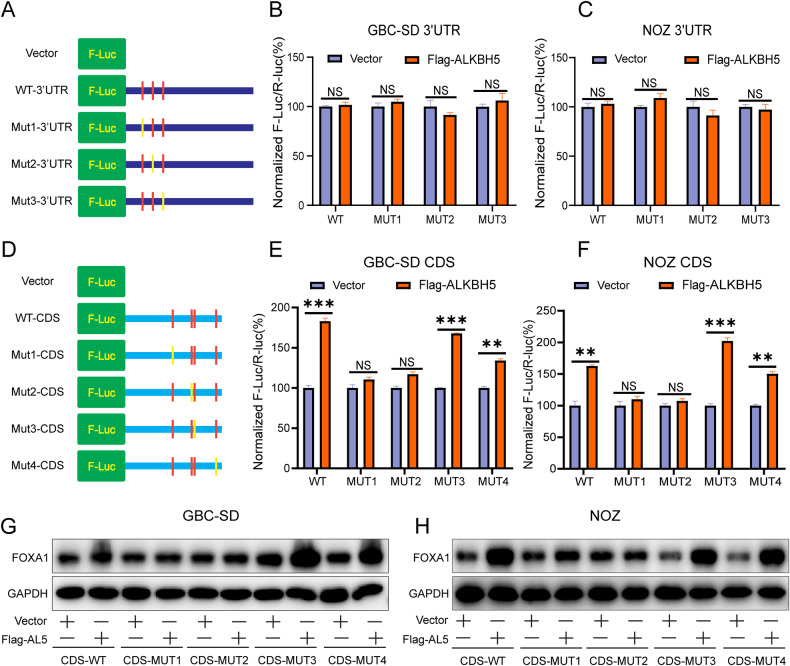
ALKBH5 promotes FOXA1 expression through site 1 and site 2. **A** Schematic diagram illustrating the construction of pmirGLO vector containing m^6^A modification sites and mutant sites in the FOXA1 mRNA 3’ UTR. **B**, **C** Dual-luciferase reporter assay measuring fluorescence values inFlag-ALKBH5-transfected GBC cells carrying pmirGLO-3’UTR (WT) or pmirGLO-3’UTR-MUT (1/2/3) after 48 h. **D** Schematic diagram illustrating the construction of plasmids containing m^6^A modification sites and mutant sites in the CDS region of FOXA1 mRNA. **E**, **F** Dual-luciferase reporter assay measuring fluorescence values in Flag-ALKBH5-transfected GBC cells carrying FOXA1-CDS-WT or FOXA1-CDS-MUT (1/2/3/4/) after 48 h. **G**, **H** Western blot analysis of FOXA1 protein changes in FlagALKBH5-transfected GBC cells carrying FOXA1-CDS-WT or FOXA1-CDS-MUT (1/2/3/4/) and corresponding vector groups after 48 h. Error bars represent the mean (n = 3) ±SEM. ^NS^*p* > 0.05, *p < 0.05, **p < 0.01, ***p < 0.001.

Next, further investigation was conducted to determine which m^6^A modification site on FOXA1 mRNA is involved in the regulation of FOXA1 expression. Firstly, the effect of m^6^A modification sites on the FOXA1 expression was studied in the 3’ UTR region of FOXA1 mRNA. Wild-type plasmid for the FOXA1 mRNA 3’ UTR (WT-3’UTR), mutant 1 plasmid for the 3’ UTR region (Mut1-3’UTR), mutant 2 plasmid for the 3’ UTR region (Mut2-3’UTR), and mutant 3 plasmid for the 3’ UTR region (Mut3-3’UTR) were constructed (Fig. 7A). Subsequently, dual-luciferase reporter assay results showed that overexpression of ALKBH5 did not alter the fluorescence values of WT-3’UTR, Mut1-3’UTR, Mut2-3’UTR, and Mut3-3’UTR groups (Fig. 7B, C). This suggests that the m^6^A modification site on the 3’ UTR region of FOXA1 mRNA does not contribute to the regulation of FOXA1 expression.

Next, the effect of m^6^A modification sites in the CDS region of FOXA1 mRNA on FOXA1 expression was investigated. Wild-type plasmid for the CDS region of FOXA1 mRNA (WT-CDS), mutant 1 plasmid for the CDS region (Mut1-CDS), mutant 2 plasmid for the CDS region (Mut2-CDS), mutant 3 plasmid for the CDS region (Mut3-CDS), and mutant 4 plasmid for the CDS region (Mut4-CDS) were constructed (Fig. 7D). Subsequently, dual-luciferase reporter assay results showed that overexpression of ALKBH5 significantly enhanced the fluorescence values of WT-CDS, Mut3-CDS, and Mut4-CDS, while having little impact on the fluorescence values of Mut1-CDS and Mut2-CDS (Fig. 7E, F). Moreover, WT-CDS, Mut3-CDS, and Mut4-CDS did not interfere with the promoting effect of Flag-ALKBH5 on FOXA1 protein, while Mut1-CDS and Mut2-CDS counteracted the promoting effect of Flag-ALKBH5 on FOXA1 protein (Fig. 7G, H). This indicates that ALKBH5 promotes FOXA1 protein expression by inhibiting m^6^A modification at site 1 and site 2 in the CDS region of FOXA1 mRNA.

### TGF-β1 inhibits the binding ability between ALKBH5 and m^6^A sites in the CDS region of FOXA1

**Fig. 8:**
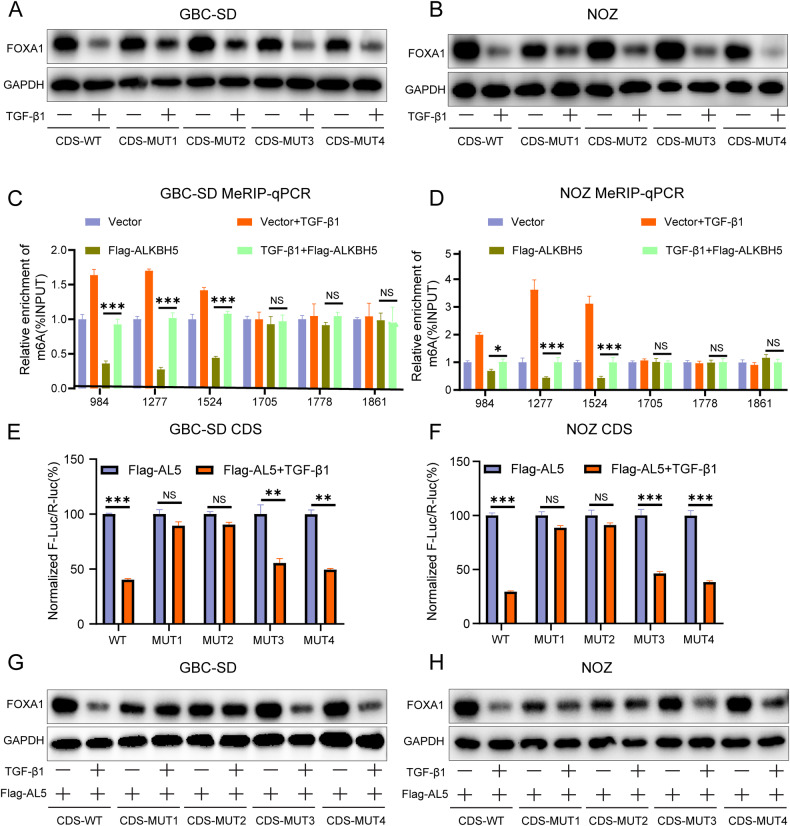
TGF-β1 inhibits the binding ability between ALKBH5 and m^6^A modification sites in FOXA1 mRNA. **A**, **B** Western blot analysis of FOXA1 protein changes in normal GBC cells transfected with FOXA1-CDS-WT or FOXA1-CDS-MUT (1/2/3/4/) with or without TGF-β1 stimulation after 48 h. **C**, **D** MeRIP qPCR assay measuring the impact of TGF-β1+Flag-ALKBH5 on m^6^A modification of FOXA1 mRNA. **E**, **F** Dual-luciferase reporter assay measuring fluorescence values in Flag- ALKBH5-transfected GBC cells carrying FOXA1-CDS-WT or FOXA1-CDS-MUT (1/2/3/4/) with or without TGF-β1 stimulation after 48 h. **G**, **H** Western blot analysis of FOXA1 protein changes in Flag-ALKBH5-transfected GBC cells carrying FOXA1-CDS-WT or FOXA1-CDS-MUT (1/2/3/4/) and corresponding vector groups with or without TGF-β1 stimulation after 48 h. Error bars represent the mean (n = 3) ±SEM. ^NS^ *p* > 0.05, *p < 0.05, **p < 0.01, ***p < 0.001.

The above results indicate that ALKBH5 promotes FOXA1 protein expression by inhibiting m^6^A modification at site 1 and site 2 in the CDS region of FOXA1 mRNA. However, it is unclear whether TGF-β1 inhibits FOXA1 protein expression through these two sites. Western blot analysis showed that WT-CDS, Mut3-CDS, and Mut4-CDS plasmids did not affect the inhibitory effect of exogenous TGF-β1 on FOXA1 protein, while Mut1-CDS and Mut2-CDS weakened the inhibitory effect of exogenous TGF-β1 on FOXA1 protein (Fig. 8A, B). This indicates that TGF-β1 inhibits FOXA1 protein expression by promoting m^6^A modification at site 1 and site 2 in the CDS region of FOXA1 mRNA.

Additionally, MeRIP showed that exogenous TGF-β1 can inhibit ALKBH5-mediated m^6^A modification in the CDS region of FOXA1 mRNA (Fig. 8C, D). Dual-luciferase reporter assay results revealed that exogenous TGF-β1 significantly decreased the fluorescence values of WT-CDS, Mut3-CDS, and Mut4-CDS groups in ALKBH5-overexpressing cells, while having little impact on the fluorescence values of Mut1-CDS and Mut2-CDS groups (Fig. 8E, F). Furthermore, exogenous TGF-β1 significantly reduced the expression of FOXA1 protein in WT-CDS, Mut3-CDS, and Mut4-CDS groups of ALKBH5-overexpressing cells, while having little effect on the expression of FOXA1 protein in Mut1-CDS and Mut2-CDS groups (Fig. 8G, H). This suggests that TGF-β1 inhibits FOXA1 protein expression by inhibiting the binding between ALKBH5 and FOXA1 mRNA, thereby promoting m^6^A modification at site 1 and site 2 in the CDS region of FOXA1 mRNA.

### FOXA1 inhibits the progression of GBC

**Fig. 9:**
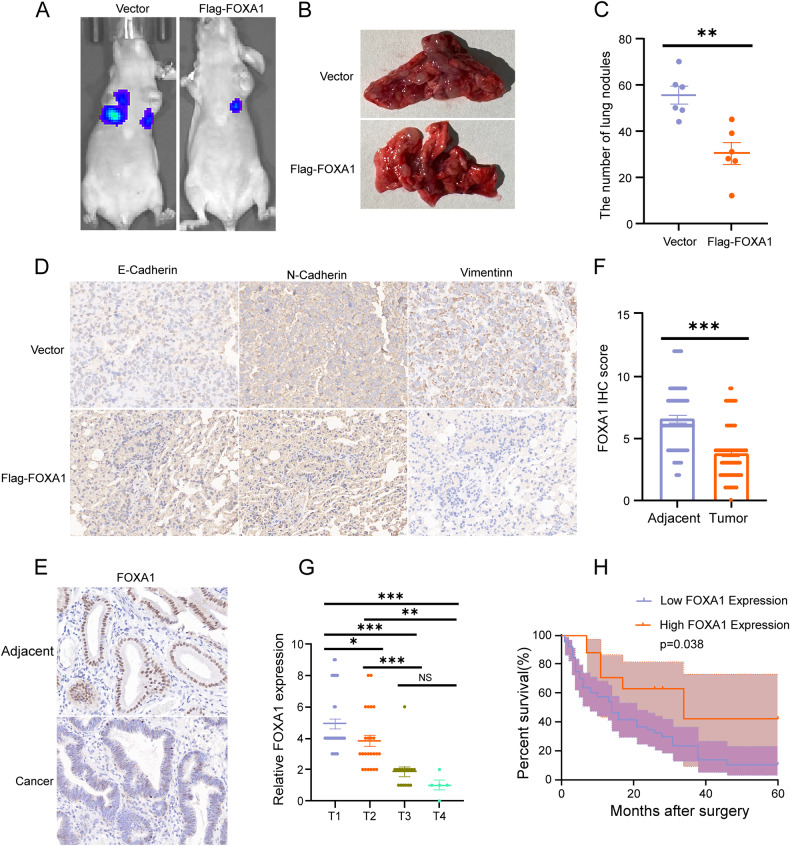
FOXA1 inhibits the progression of GBC. **A** Fluorescence imaging of lung metastasis in nude mice injected with GBC cells overexpressing FOXA1 (Flag-FOXA1 group) and corresponding vector cells. **B**, **C** Effect of FlagFOXA1 on the number of metastatic nodules in GBC lung metastasis. **D** Expression of E-Cadherin, N-Cadherin, and Vimentin in the lung metastatic tumor model of GBC. **E**, **F** Expression of FOXA1 in GBC tissues and corresponding adjacent tissues. **G** Expression of FOXA1 in different stages of GBC. **H** Relationship between FOXA1 expression and overall survival rate in GBC patients. Error bars represent the mean (n = 3) ±SEM. ^NS^*p* > 0.05, *p < 0.05, **p < 0.01, ***p < 0.001.

NOZ cells overexpressing FOXA1 and the corresponding vector cells were used to establish a nude mouse pulmonary metastasis model through tail vein injection to evaluate the role of FOXA1 in GBC metastasis. The results showed that the pulmonary metastasis was significantly weaker in the FOXA1 overexpression group compared to the corresponding vector group (Fig. 9A–C). Immunohistochemical analysis of lung transplant tumor paraffin sections revealed that the expression level of E-cadherin was significantly increased, while the expression levels of N-cadherin and vimentin were significantly decreased in the lung metastatic tissues of the Flag-FOXA1 group compared to the vector group (Fig. 9D). Overall, these findings indicate that FOXA1 can significantly inhibit the migration ability of GBC.

To demonstrate the correlation between FOXA1 expression and clinical pathological features in GBC patients, immunohistochemical analysis was performed to assess the protein expression level of FOXA1 in 80 cases of GBC tissues and corresponding adjacent tissues. The results showed that FOXA1 expression in the cancer tissues was weaker than that in the adjacent tissues of GBC clinical specimens (Fig. 9E, F). FOXA1 expression was negatively correlated with distant metastasis and TNM staging, but showed no significant correlation with gender, age, or the presence of gallstones (Table 3). Moreover, FOXA1 expression gradually decreased from T1 to T4 stage of GBC (Fig. 9G). Additionally, Kaplan-Meier survival analysis revealed that patients with high FOXA1 expression had a higher overall survival rate compared to those with low FOXA1 expression (Fig. 9H). In conclusion, FOXA1 can inhibit the progression of GBC.

**Table 3:** Expression of FOXA1 in GBC specimens and its correlation with clinicopathological parameters.

		Protein expression of FOXA1		
Clinicopathological features	Case	Low (n = 63)	High (n = 17)	*P*
Age (years)				0.810
<60 years old	45	35	10	
≥60 years old	35	28	7	
Gender				0.109
Male	38	27	11	
Female	42	36	6	
TNM stage				0.032*
I–II	59	43	16	
III–IV	21	20	1	
Distant metastasis				0.010**
(+)	41	37	4	
(−)	39	26	13	
Gallstone				0.503
(+)	48	39	9	
(−)	32	24	8	

Low FOXA1 expression is scored 0–4.

High FOXA1 expression is scored 6–12.

Bold value indicates significant differences between groups.

*P＜0.05, **P＜0.01.

## Discussion

Recent research indicates that m^6^A modification is currently the most common mRNA modification in human cancer development, with significant implications for the diagnosis, treatment, and prognosis of malignant tumors [47]. m^6^A modification alters the expression levels of oncogenes or tumor suppressor genes by regulating post-transcriptional modifications, impacting multiple processes in tumorigenesis including cell proliferation, EMT, angiogenesis, and therapeutic resistance [48–51]. Our results demonstrate that TGF-β1 inhibits the expression of FOXA1 protein in GBC. FOXA1 can suppress TGF-β1-induced migration and EMT of GBC cells. Furthermore, TGF-β1 promotes m^6^A modification in the CDS region of FOXA1 mRNA by inhibiting the expression of ALKBH5, leading to reduced translation efficiency of FOXA1 mRNA. It has also been confirmed that TGF-β1 can inhibit the binding ability of ALKBH5 to m^6^A sites in the FOXA1 CDS region.

The ability of tumor cells to undergo EMT is one of the key factors affecting the metastasis of malignant tumors [52]. Therefore, the metastasis of GBC is closely related to the EMT capacity of GBC cells. Our study comprehensively examined the EMT functional indicators after stimulation by TGF-β1. According to previous research reports, there is controversy regarding the relationship between TGF-β1 and FOXA1. Our research found a negative correlation between the expression of TGF-β1 and FOXA1 in GBC pathological tissues and fresh frozen tissues. Furthermore, in GBC cell lines, TGF-β1 can inhibit FOXA1 protein expression. Therefore, our study establishes a negative correlation between TGF-β1 and FOXA1, demonstrating from both endogenous and exogenous perspectives that TGF-β1 inhibits FOXA1 protein expression. Additionally, this study revealed for the first time that FOXA1 can inhibit the EMT capacity and migration ability of GBC cells by promoting the expression of E-cadherin and suppressing the expression of N-cadherin, vimentin, Snail, and Slug, both in in vivo and in vitro models of GBC. Moreover, in vitro rescue experiments further confirmed that ectopic expression of FOXA1 reversed the migration and EMT effects mediated by TGF-β1 in GBC cells. Furthermore, patients with high FOXA1 expression in clinical specimens of GBC had a higher overall survival rate compared to those with low FOXA1 expression. This suggests that FOXA1 can serve as an important prognostic indicator for GBC. Subsequent studies further explored the molecular mechanisms behind TGF-β1’s inhibition of FOXA1 protein expression in GBC.

Interestingly, our research revealed that using the SMAD2 phosphorylation inhibitor AZ12601011 only slightly mitigates the inhibition of FOXA1 protein expression by TGF-β1. This suggests that the p-SMAD2 pathway is not a major mechanism for TGF-β1’s inhibitory effect on FOXA1 protein expression in GBC cells. Additionally, the introduction of the p21 inhibitor UC2288 and the c-Myc inhibitor 10058-F4 did not significantly counteract the inhibition of FOXA1 protein expression by TGF-β1, indicating that the cell cycle arrest induced by TGF-β1 does not significantly affect FOXA1 protein expression in these cells. We also found that in GBC cells, under the same concentration of TGF-β1, the expression of FOXA1 mRNA was unaffected. Results from polysome profiling analysis demonstrated that TGF-β1 can reduce the levels of FOXA1 mRNA in the translationally active polysome fraction. Translational regulation plays a pivotal role in gene expression, accounting for more than half of all regulatory activities and serving as the most critical step in cellular processes [53]. Consequently, even a slight reduction in translational activity might lead to a significant decrease in protein production. This study provides innovative insights into the molecular mechanism by which TGF-β1 inhibits the expression of FOXA1 protein in GBC, focusing on the transcription and translation of FOXA1. Furthermore, it suggests the potential role of TGF-β1 in regulating the translation efficiency of downstream target gene mRNAs.

Research has shown that m^6^A modification can dynamically regulate mRNA translation efficiency to control protein expression levels [29, 54–58]. In neuronal axons, specific inhibition or knockdown of the m^6^A demethylase FTO promotes m^6^A modification of GAP-43 mRNA, leading to the suppression of local translation of GAP-43 and inhibition of axon elongation [59]. In adipose tissue, knockdown of FTO increases m^6^A modification of Angptl4 mRNA, reducing Angptl4 translation and altering triglyceride metabolism [60]. To date, there have been no reports on m^6^A modification of FOXA1 mRNA. In our study, we found that treatment of GBC cells with the FTO-targeting inhibitor FB23 and the m^6^A methyltransferase METTL3-targeting inhibitor STM2457 showed that FB23 does not alter the protein levels of FOXA1, while STM2457 significantly increases FOXA1 protein levels. Furthermore, the addition of STM2457 can counteract the inhibitory effect of TGF-β1 on FOXA1 protein expression. This finding suggests that m6A modification plays a significant role in regulating FOXA1 protein expression in GBC cells, highlighting its importance in cellular regulation. This research could potentially lay the groundwork for the future development and use of m^6^A-specific inhibitors in malignancies characterized by high FOXA1 expression, providing a theoretical basis for targeted therapeutic strategies. Through MeRIP experiments, the results show that TGF-β1 promotes m^6^A modification of the FOXA1 mRNA CDS region, without affecting m^6^A modification in the 3’ UTR of FOXA1 mRNA. In summary, our study confirms that TGF-β1 diminishes FOXA1 mRNA translation efficiency through alterations in m^6^A modification. Furthermore, in vivo experiments demonstrated that TGF-β1 accelerates the growth of subcutaneous xenograft tumors in nude mice. While Zugmaier et al. reported potential systemic effects of TGF-β1 like cachexia and fibrosis, our study did not observe significant signs of these conditions in the TGF-β-treated cohort. This discrepancy may indicate a divergence in biological responses based on the model system or experimental conditions. These findings underscore TGF-β1’s role in facilitating the malignant progression of GBC. Previous studies [61–63] have shown significant therapeutic benefits from targeting TGF-β1 in various tumors, suggesting that blocking TGF-β1 could be a promising new direction for targeted therapy in GBC. Although our study did not pursue targeted therapy against TGF-β1, we plan to explore this therapeutic approach in future research to potentially enhance GBC treatment strategies.

In recent years, m^6^A modification has garnered strong attention in the study of GBC. In GBC, deoxycholic acid (DCA) disrupts the METTL14-METTL3-WTAP complex by binding to METTL3, inhibiting m^6^A modification of the precursor pri-miR-92b of miR-92b-3p, and significantly reducing the progression rate from pri-miR-92b to its mature form [64]. In another study, IGF2BP3 was found to activate the PAR2/AKT axis, enhancing the invasive ability of GBC, by promoting KLK5 mRNA stability in an m^6^A-dependent manner [65]. Additionally, high expression of METTL3 in GBC was shown to mediate m^6^A modification of DUSP5 mRNA, promoting proliferation, invasion, and migration of GBC [66]. In our study, we examined the changes in the major components of the m^6^A methyltransferase complex, METTL3/METTL14/WTAP, as well as the demethylases ALKBH5 and FTO, in GBC cells after TGF-β1 stimulation. The results suggest that TGF-β1 may promote m^6^A modification of FOXA1 mRNA by inhibiting the expression of the m^6^A demethylase ALKBH5. RIP, MeRIP, and point mutation experiments at the binding sites demonstrated that ALKBH5 promotes FOXA1 protein expression by inhibiting m^6^A modification at site 1 and site 2 in the CDS region of FOXA1 mRNA. Rescue experiments further confirmed that overexpression of ALKBH5 could attenuate the inhibitory effect of TGF-β1 on FOXA1 protein. This study provides the first evidence of the involvement of ALKBH5 in the process of TGF-β1-mediated suppression of FOXA1 protein expression and confirms that TGF-β1 promotes m^6^A modification in the FOXA1 CDS region by inhibiting ALKBH5 expression, leading to decreased translation efficiency of FOXA1 mRNA. Furthermore, it was demonstrated that TGF-β1 can suppress the binding ability of ALKBH5 to m^6^A sites 1 and 2 in the FOXA1 CDS region. These findings elucidate the closely related relationship between TGF-β1 and m^6^A modification of FOXA1 mRNA, as well as the molecular mechanism by which TGF-β1 suppresses FOXA1 protein expression, providing strong evidence for further understanding TGF-β1-mediated regulation of m^6^A modification.

However, the intricate nature of m^6^A modification in regulating mRNA translation efficiency necessitates further investigation to fully understand how m^6^A modification within the CDS region of FOXA1 reduces translation efficiency. Current research by He et al. indicates that the influence of m^6^A on translation efficiency is notably variable and depends on the interaction with specific RNA-binding proteins [54]. Moreover, it is suspected that there could be additional m^6^A reader proteins yet to be discovered, each potentially exerting unique effects on the translation process after m^6^A modification [54]. Another study proposed that m^6^A modification of mRNA may hinder the binding and translation elongation process of tRNA-protein complexes [67], further suggesting that the regulation of translation efficiency by m^6^A modification will be a major research focus and challenge in molecular biology in the future. By balancing the interplay between m^6^A modification and translation efficiency, there will be broad applications in drug development and disease treatment. In summary, in this study, we have provided convincing in vitro and in vivo evidence to demonstrate that TGF-β1 can regulate FOXA1 translation efficiency and promote GBC migration and EMT progression through m^6^A modification. This provides novel insights into tumor metastasis and offers some assistance in the research of targeted therapies for cancer.

### Supplementary information

Supplementary Material

Original western blots

## Data Availability

The datasets used and analyzed during the current study are available from the corresponding author on reasonable request.
