# Supplementary material for: TGF-β1 facilitates gallbladder carcinoma metastasis by regulating FOXA1 translation efficiency through m6A modification
Source: Cell Death Dis. 2024 Jun 17;15(6):422. doi: 10.1038/s41419-024-06800-9 (PMC11183149; doi:10.1038/s41419-024-06800-9)
Supplement: Supplementary file 1 — Supplementary Material [file 41419_2024_6800_MOESM1_ESM.pdf]

Supplementary Figure legends

Figure S1. The effects of FOXA1 knockdown on GBC cell migration and EMT. (A) Western blot analysis of the knocking down efficiency of FOXA1. (B-C) Transwell assay to determine the changes in the migratory capacity of GBC cells after knocking down FOXA1. (D-E) Wound healing assay to assess the changes in the migratory capacity of GBC cells after knocking down FOXA1. (F) Western blot analysis to determine the changes in the protein levels of EMT-related markers in GBC cells after knocking down FOXA1. Error bars represent the mean (n=3) ± SEM. $^{NS}p>0.05$, $*p<0.05$, $**p<0.01$, $***p<0.001$.

Figure S2. The role of cell cycle and p-Smad2 pathway in TGF-β1 induced inhibition of FOXA1 protein expression. (A) The effect of AZ12601011 on TGF-β1 inhibition of FOXA1 protein. (B) The effect of UC2288 or 10058-F4 on TGF-β1 induced inhibition of FOXA1 protein expression.

Figure S3. The effect of the METTL3 inhibitor STM2457 on TGF-β1 induced inhibition of FOXA1 protein expression.

Figure S4. The binding of ALKBH5 to the m^6A modification site of FOXA1 mRNA was detected by RIP assay.

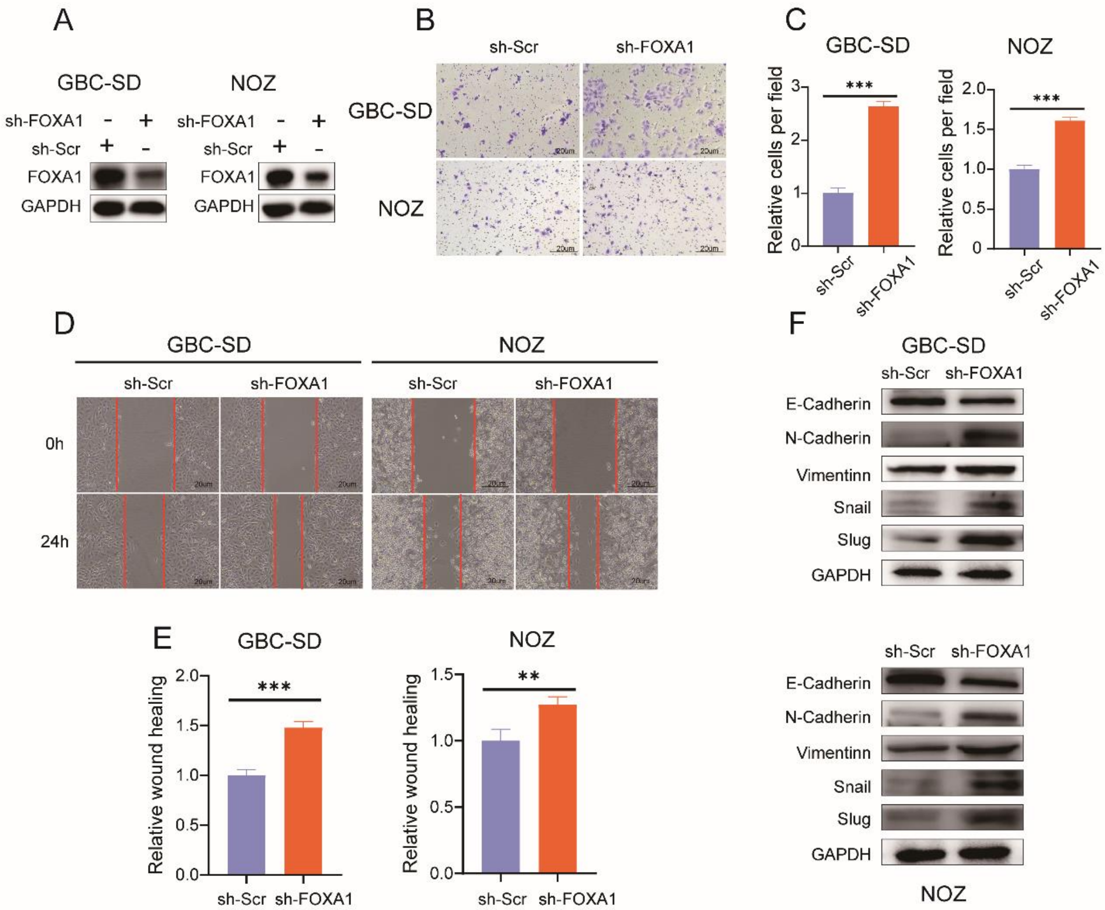
A
GBC-SD
NOZ
sh-FOXA1 - +
sh-Scr + -
FOXA1
GAPDH
B
sh-Scr
sh-FOXA1
GBC-SD
NOZ
C
GBC-SD
NOZ
Relative cells per field
***
sh-Scr
sh-FOXA1
D
GBC-SD
NOZ
sh-Scr
sh-FOXA1
0h
24h
E
GBC-SD
NOZ
Relative wound healing
***
**
sh-Scr
sh-FOXA1
F
GBC-SD
sh-Scr
sh-FOXA1
E-Cadherin
N-Cadherin
Vimentinn
Snail
Slug
GAPDH
NOZ

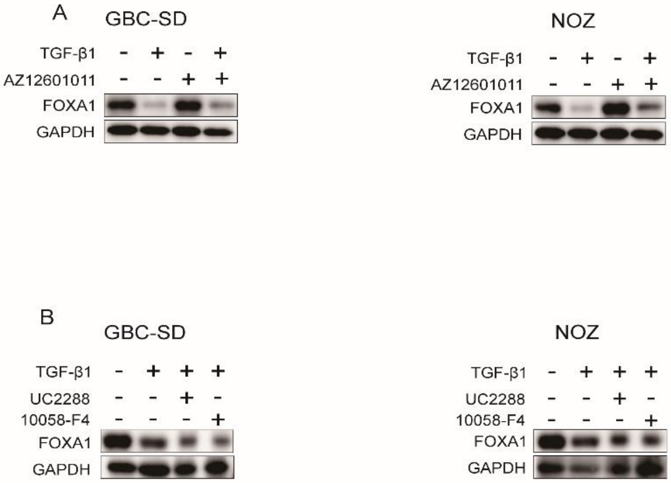
A
GBC-SD
TGF-β1
AZ12601011
FOXA1
GAPDH
NOZ
TGF-β1
AZ12601011
FOXA1
GAPDH
B
GBC-SD
TGF-β1
UC2288
10058-F4
FOXA1
GAPDH
NOZ
TGF-β1
UC2288
10058-F4
FOXA1
GAPDH

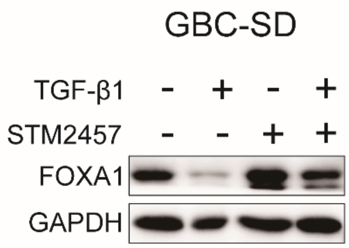
GBC-SD
TGF-β1 - + - +
STM2457 - - + +
FOXA1
GAPDH

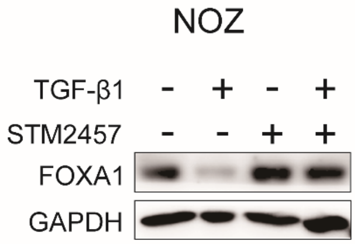
NOZ
TGF-β1 - + - +
STM2457 - - + +
FOXA1
GAPDH

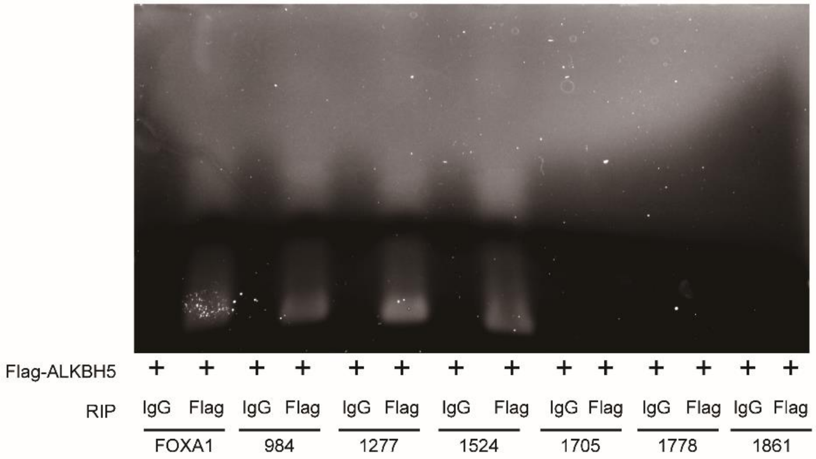
Flag-ALKBH5
+ + + + + + + + + + + + + +
RIP
IgG Flag IgG Flag IgG Flag IgG Flag IgG Flag IgG Flag IgG Flag
FOXA1
984
1277
1524
1705
1778
1861
