# Supplementary figures and images for: TGF-β1 facilitates gallbladder carcinoma metastasis by regulating FOXA1 translation efficiency through m6A modification
Source: Cell Death Dis. 2024 Jun 17;15(6):422. doi: 10.1038/s41419-024-06800-9 (PMC11183149; doi:10.1038/s41419-024-06800-9)

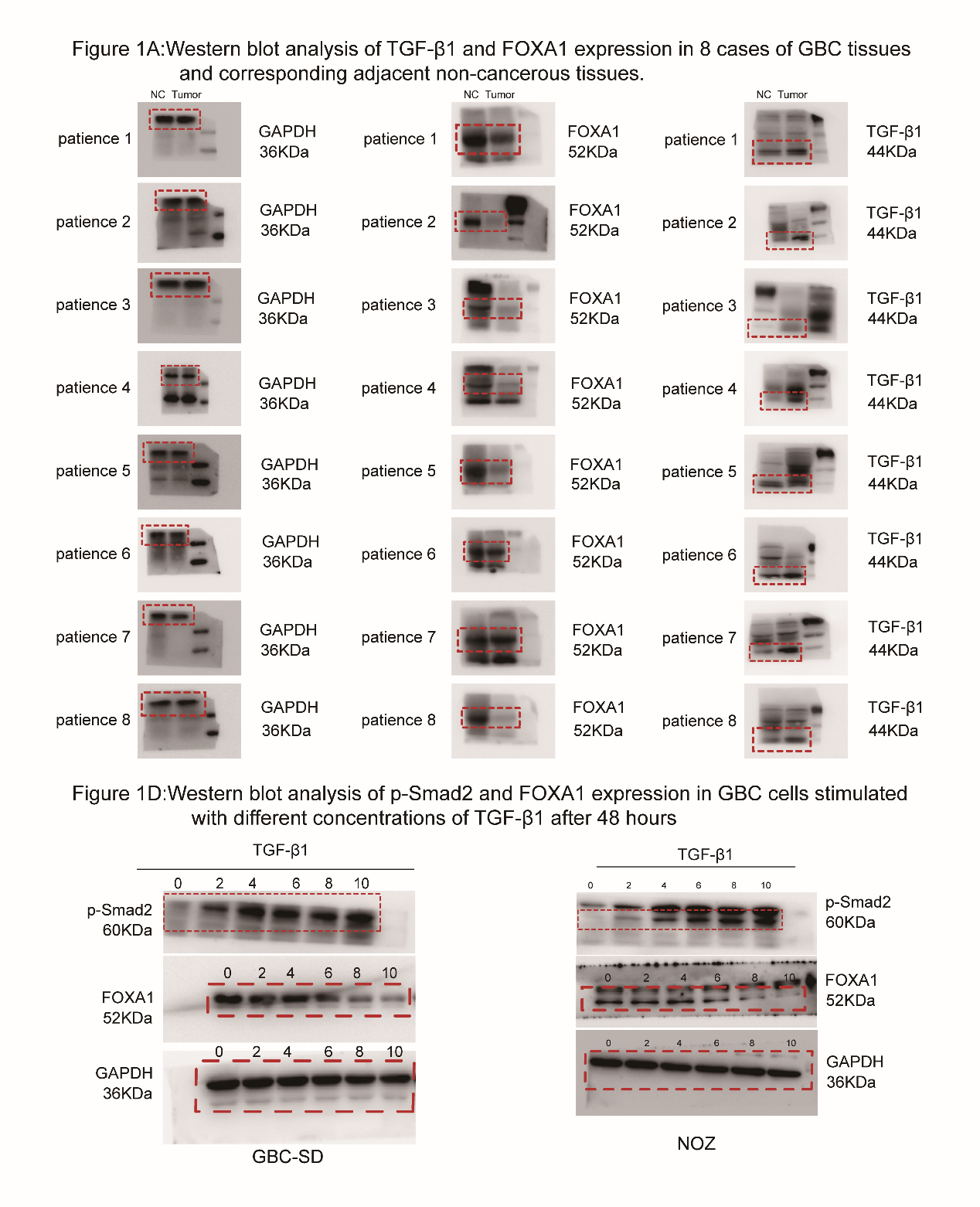

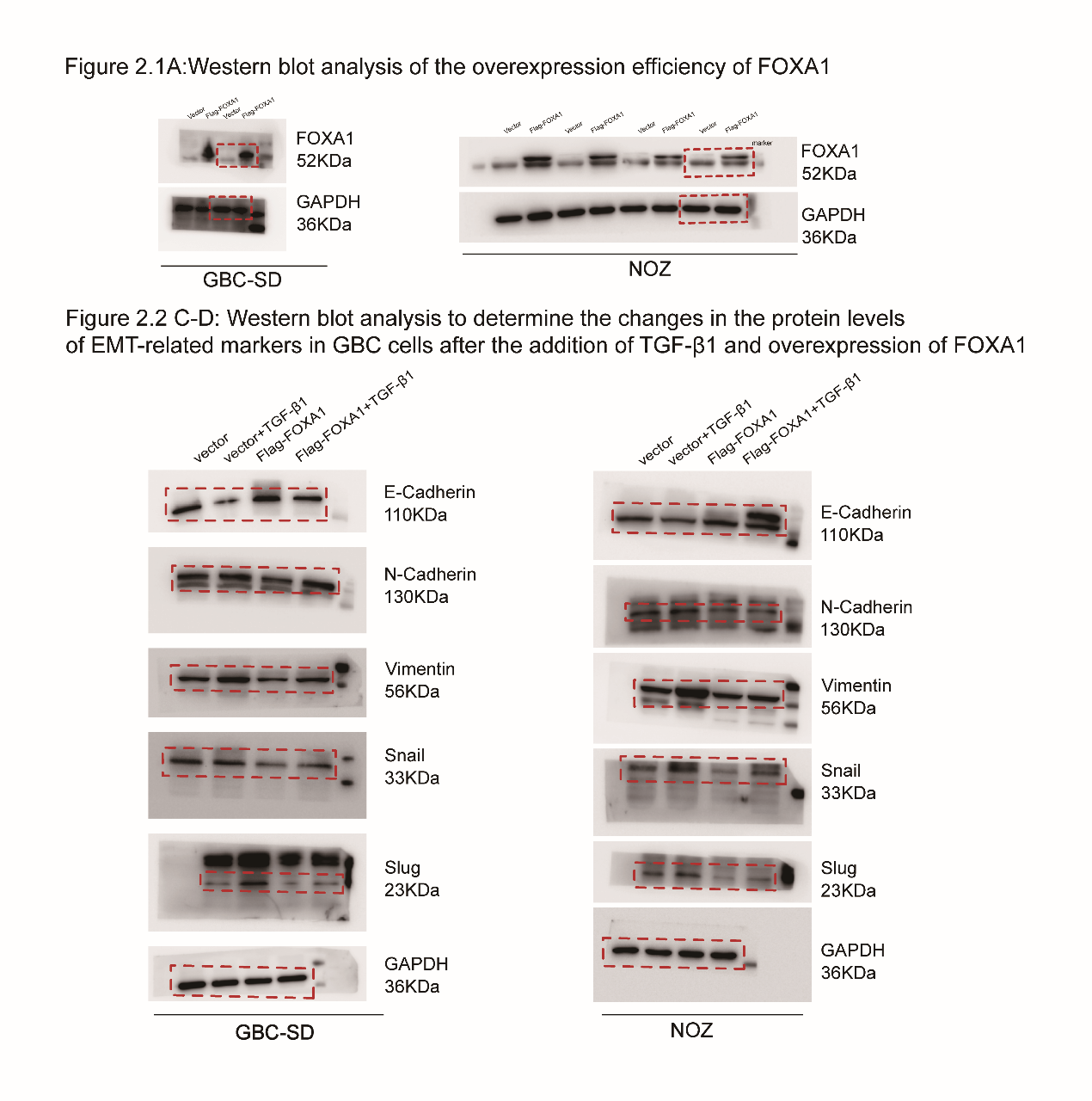

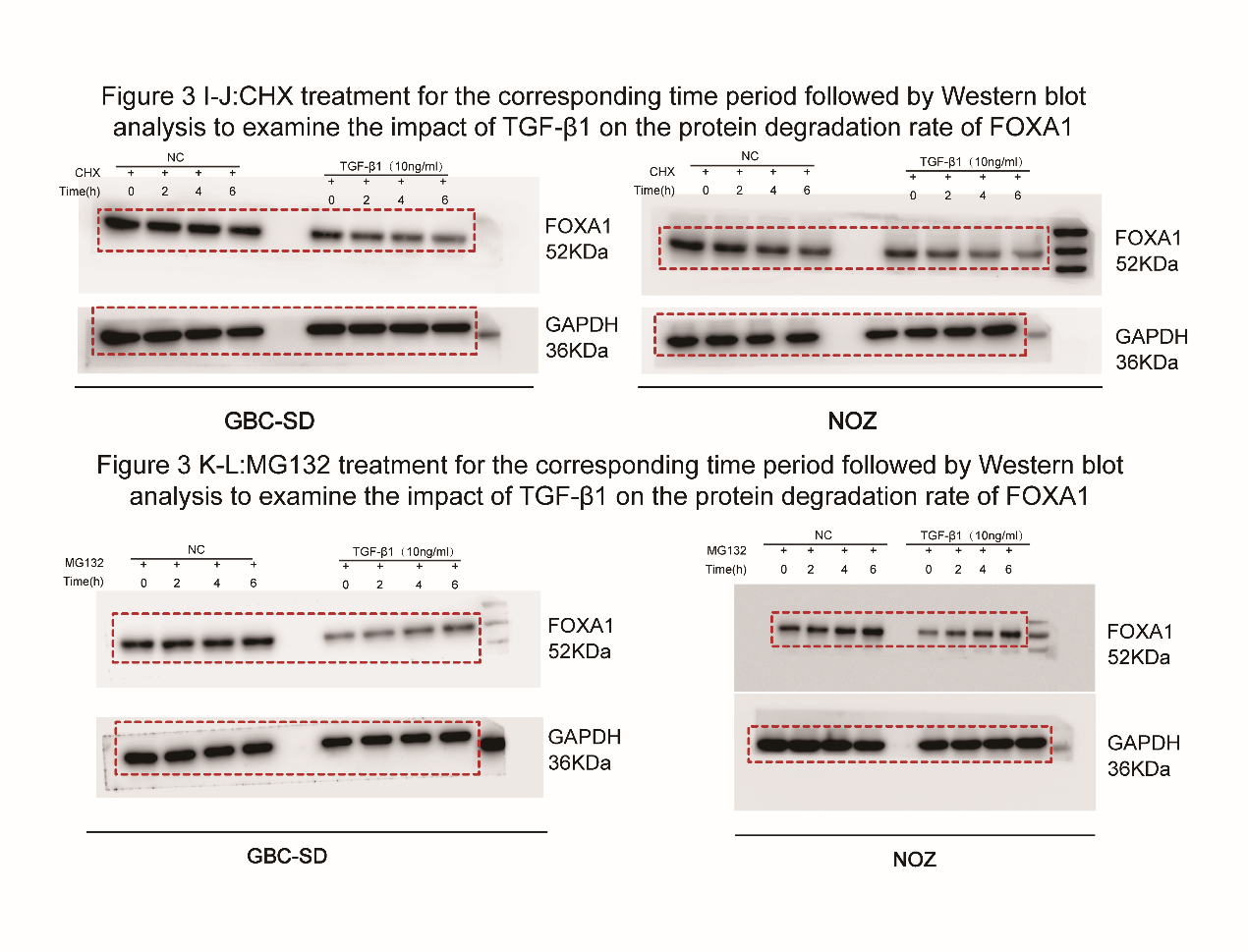

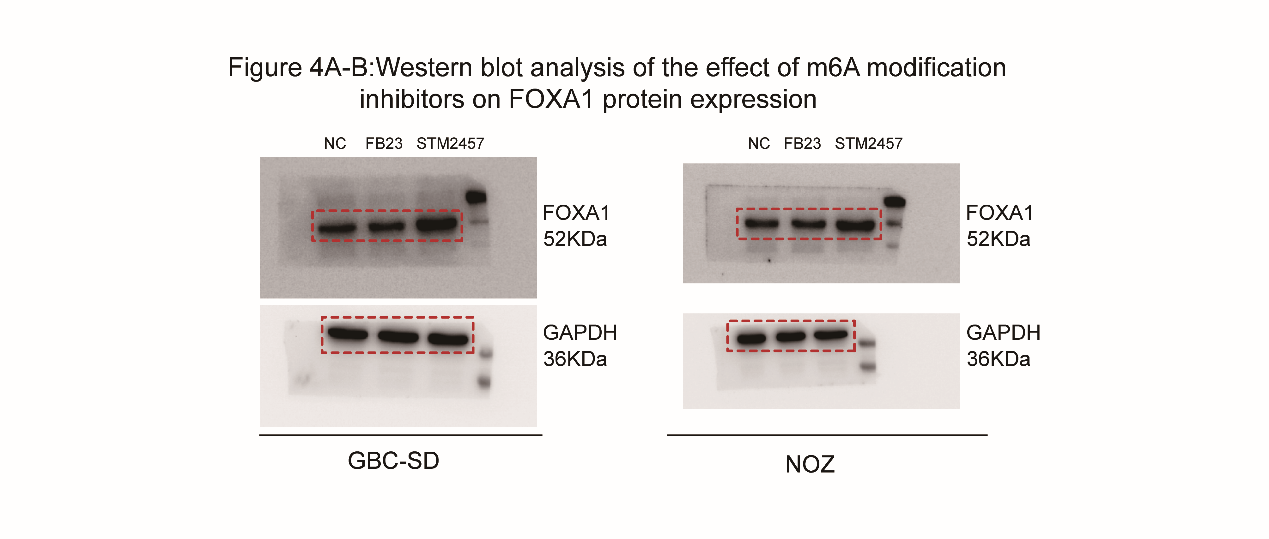

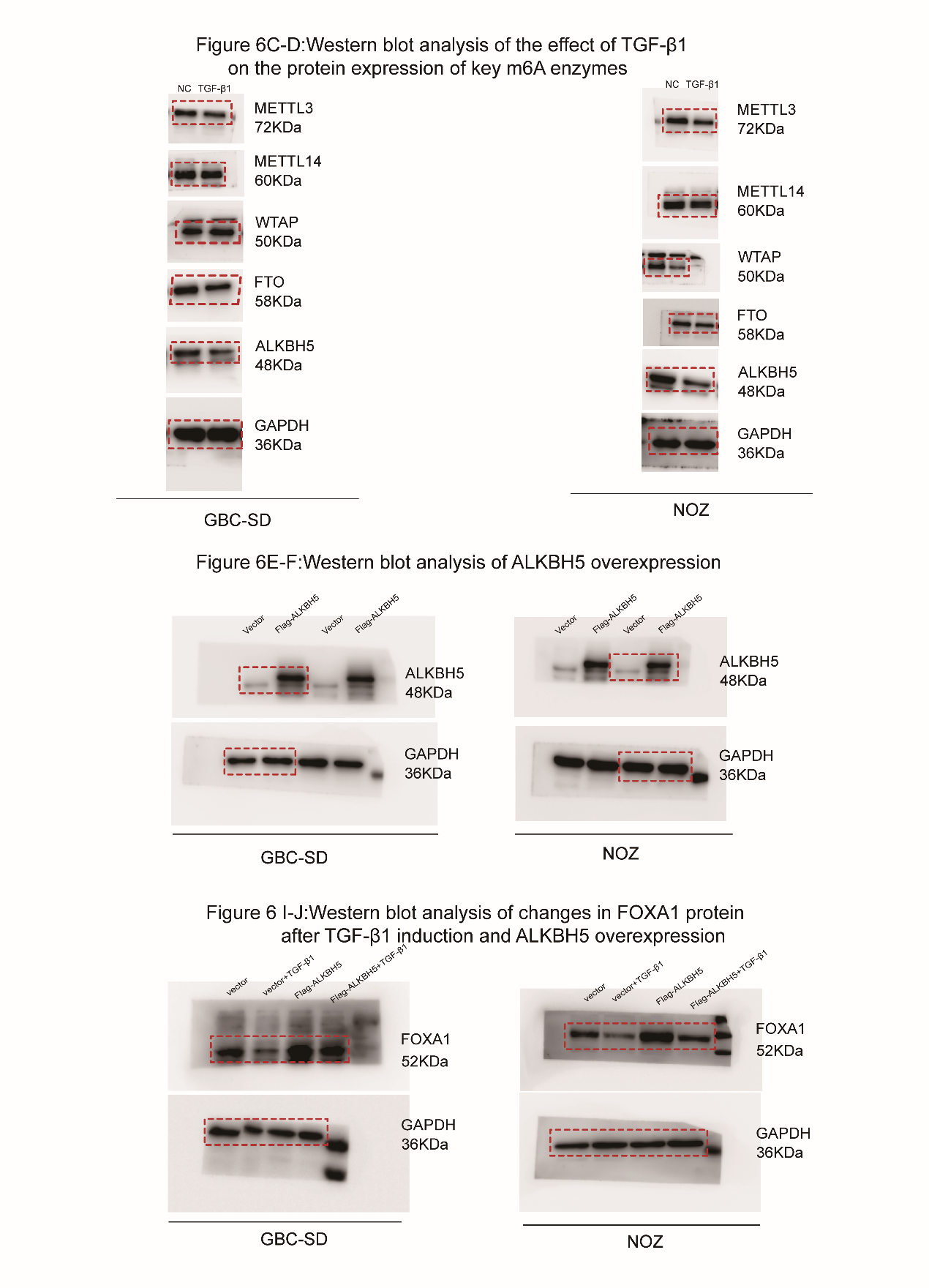

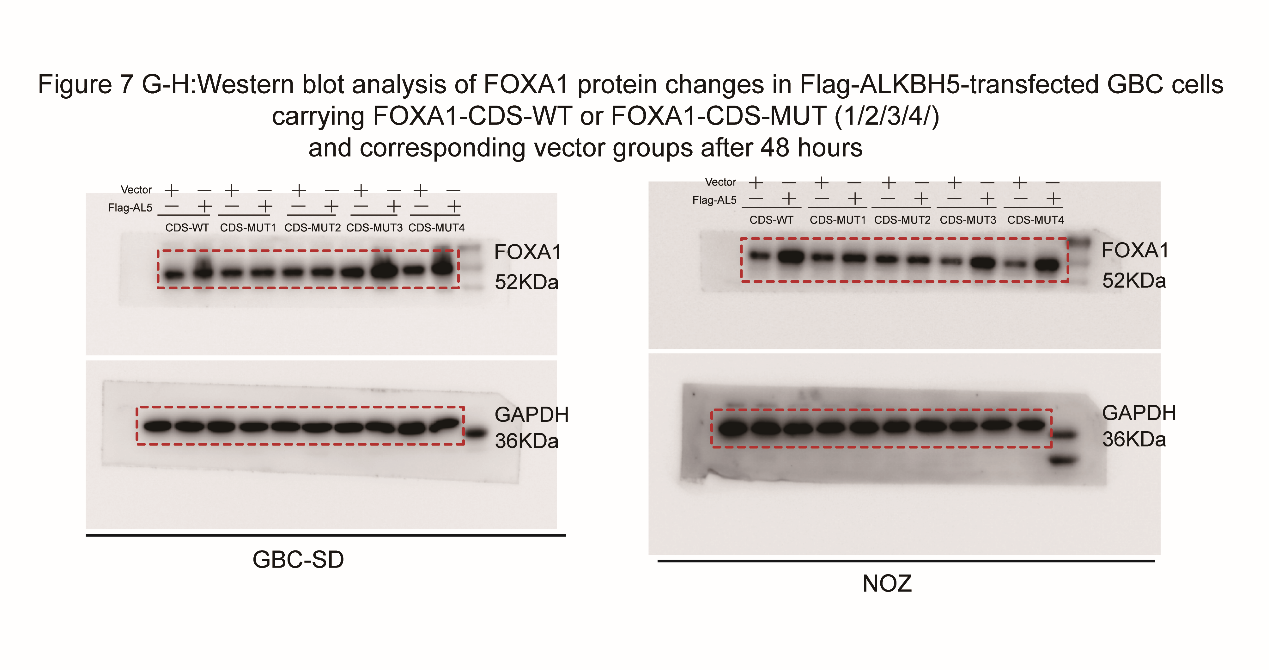

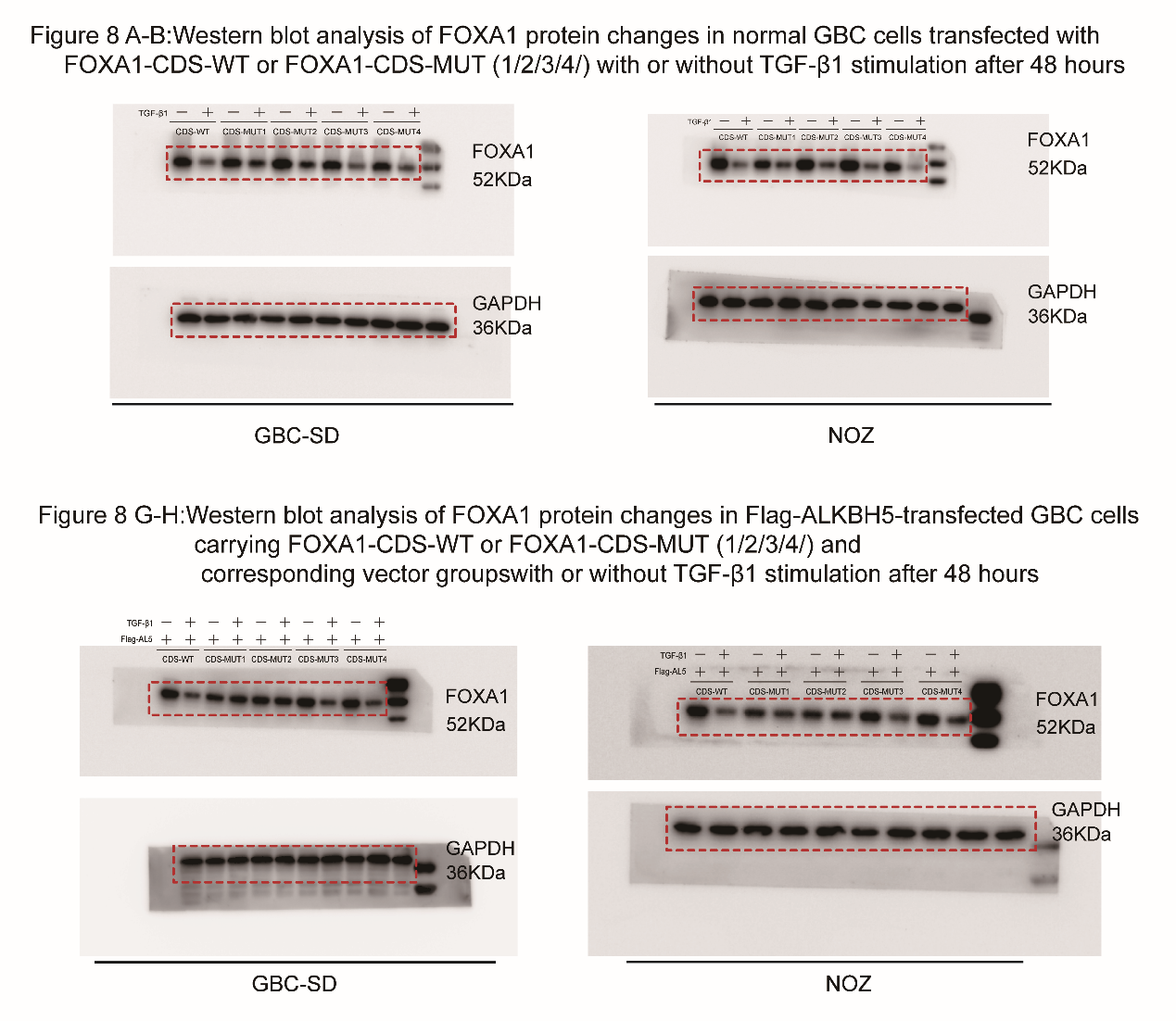

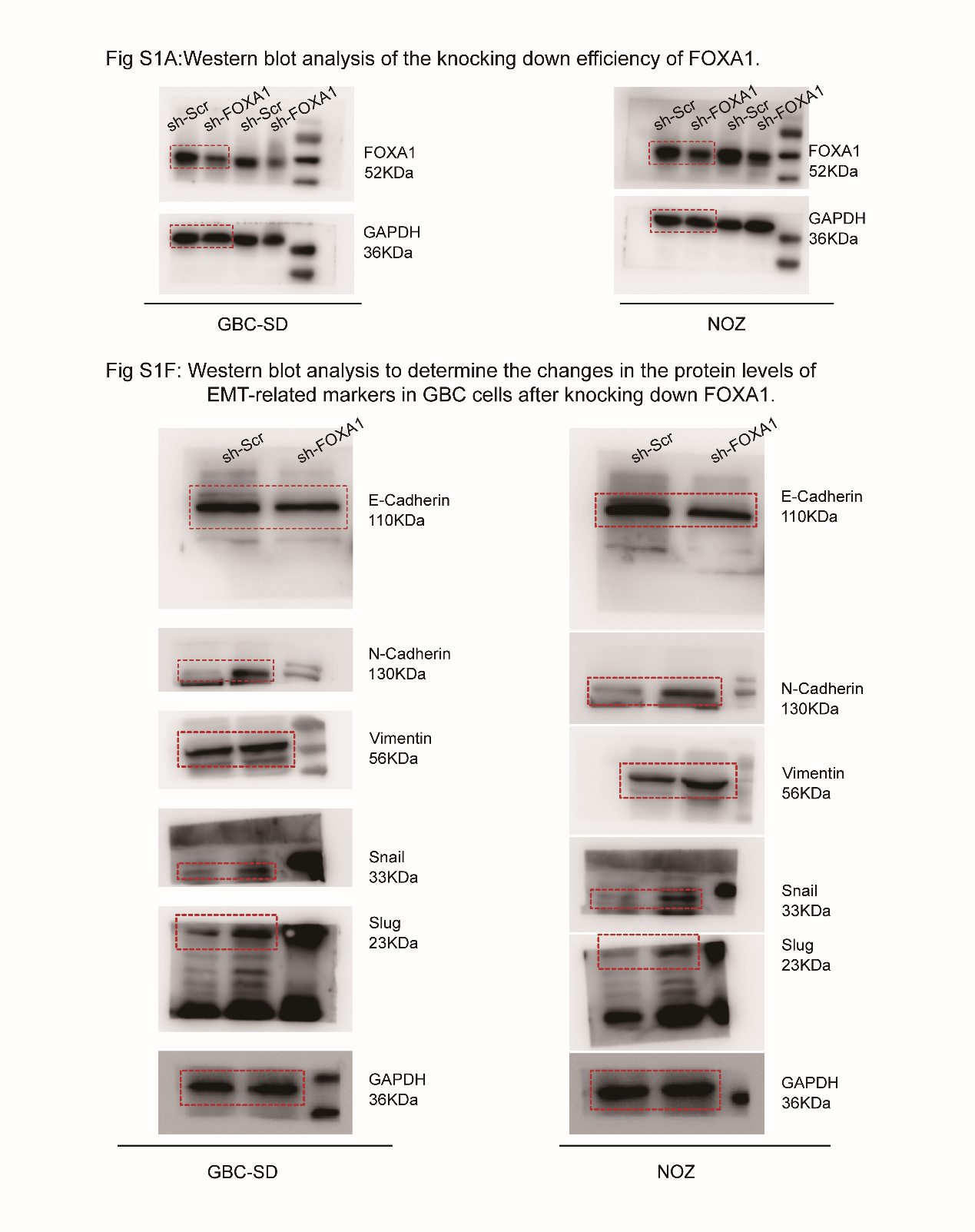

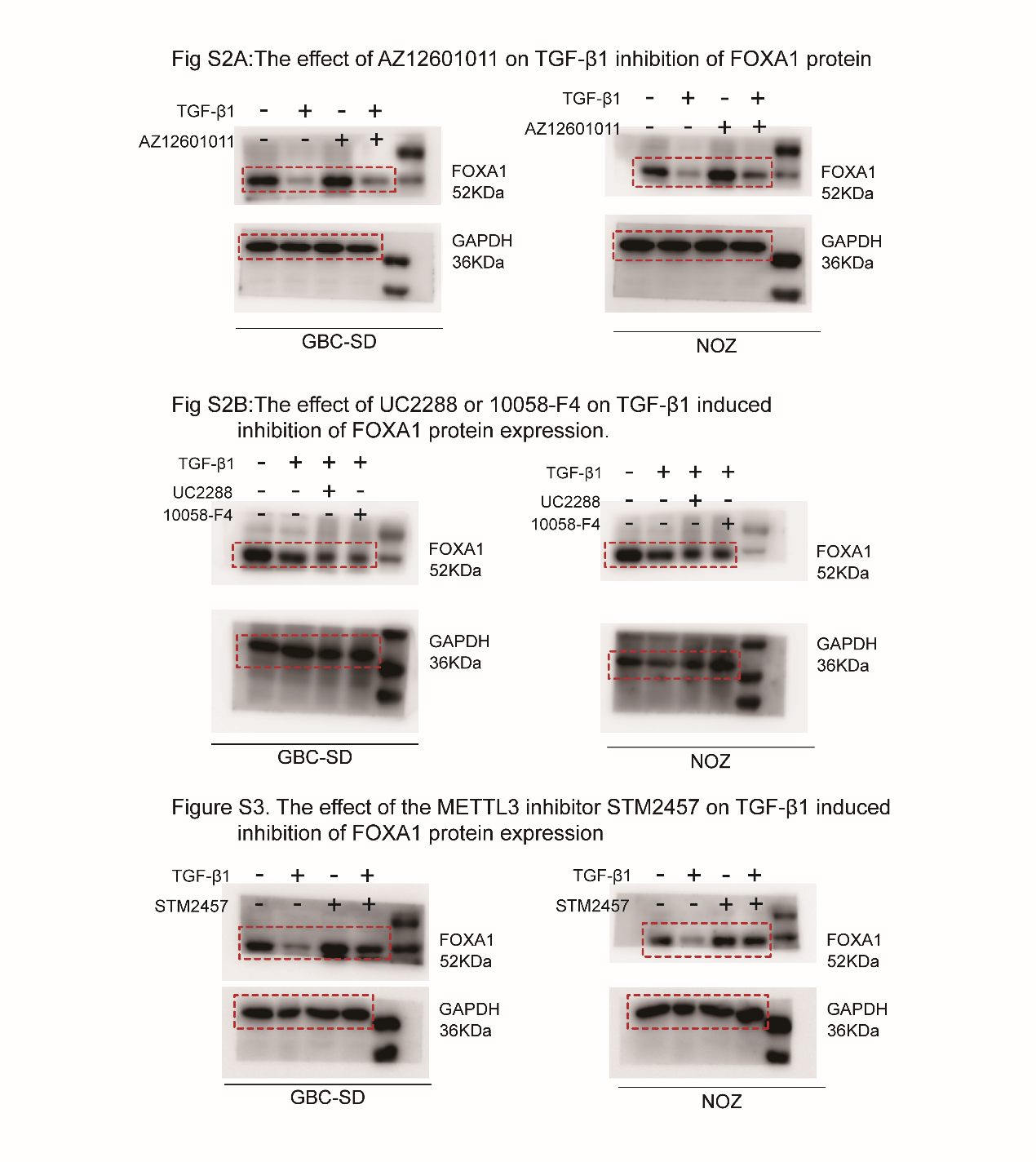

Supplement: Supplementary file 2 — Original western blots [file 41419_2024_6800_MOESM2_ESM.docx]
